# Global burden of urticaria in children and adolescents, 1990 to 2021: Trends, disparities, and future projections

**DOI:** 10.1097/MD.0000000000047488

**Published:** 2026-01-30

**Authors:** Songhui Liu, Xiaohui Jing, Fucheng Feng, Honglin Wang, Ting He

**Affiliations:** a School of Nursing, Guangzhou Huali College, Guangzhou, Guangdong Province, People’s Republic of China; b School of Nursing, Southwest Jiaotong University Hope College, Chengdu, Sichuan, People’s Republic of China; c Department of Professional Nursing Studies, Kulliyyah of Nursing, International Islamic University Malaysia, Kuala Lumpur, Malaysia.

**Keywords:** burden of disease, children and adolescents, GBD 2021, urticaria

## Abstract

This study aimed to analyze the global, regional, and national burden of urticaria among individuals under 20 years old using 2021 global burden of disease data. Data on prevalence, incidence, and disability-adjusted life-years (DALYs) of urticaria in children and adolescents from 1990 to 2021 were extracted. The estimated annual percentage change was calculated to assess temporal trends. The sociodemographic index (SDI) was used to evaluate regional differences, and Bayesian age-period-cohort modeling was applied to predict future trends. From 1990 to 2021, global age-standardized prevalence (ASPR), incidence (ASIR), and DALY rates of urticaria showed marginal changes. In 2021, ASPR was 1203.16 per 100,000, ASIR was 2142.29 per 100,000, and DALY rate was 73.75 per 100,000. The estimated annual percentage change was positive for all 3 indicators: 0.035 for ASPR, 0.024 for ASIR, and 0.048 for DALY rate. Females had higher rates than males. The highest age-standardized indicators were in low and middle SDI regions, with Central Europe having the highest rates and Western Europe the lowest. Prevalence peaked in children under 5 years old and decreased with age. Future projections indicate a gradual increase in the burden of urticaria by 2050. Urticaria is a significant health issue among children and adolescents, with notable disparities. Targeted prevention and early intervention, particularly in low- and middle-SDI regions, alongside strengthened global policies to ensure effective diagnosis and treatment, are imperative to mitigate its future burden.

## 1. Introduction

Urticaria, a prevalent skin condition driven by cutaneous vasodilation and increased vascular permeability, manifests as wheals and angioedema.^[1]^ Although children and adolescents are disproportionately affected, the exact prevalence and comprehensive disease burden in this demographic are poorly understood, representing a significant epidemiologic void that warrants further investigation.

Urticaria, especially chronic urticaria, has a profound impact on the physical and mental health of children and adolescents. Studies have shown that patients with chronic spontaneous urticaria (CSU) have a higher risk of comorbid psychiatric, allergic, and autoimmune disorders.^[2,3]^ Although this disease is nonfatal, children with chronic urticaria have higher absenteeism rates, poorer academic performance, and lower quality of life compared with children suffering from other allergic diseases.^[4]^ Among adolescents, patients with CSU have 3.5 times, 1.7 times, and 1.6 times higher risks of sleep disorders, anxiety, and depression, respectively, compared with the general population.^[5]^ Chronic urticaria is also associated with significant impairment in both work and nonwork activities, as well as a substantial increase in the use of healthcare resources.^[6]^

Over the past few decades, the global burden of urticaria in children and adolescents has increased. However, longitudinal tracking of this burden across multiple regions remains limited. The global burden of disease (GBD) study offers a standardized approach to describe health loss epidemiology across age groups. This research updates and extends previous analyses, focusing on prevalence, incidence, and disability-adjusted life-years (DALYs) of urticaria in individuals under 20 years from 1990 to 2021. A Bayesian age-period-cohort (BAPC) model was used to predict trends up to 2050. We further hypothesized that the burden would vary significantly by socio-demographic index (SDI), with a higher prevalence and DALY rate anticipated in lower SDI regions.

## 2. Method

### 2.1. Overview and data collection

This study utilized GBD 2021 data to analyze age-specific urticaria burden in children and adolescents. GBD 2021 provides epidemiological estimates for 371 diseases and injuries across 21 regions and 204 countries from 1990 to 2021. In this survey, we extracted data specific to urticaria in children and adolescents, including prevalence, incidence, and DALYs. DALYs combine years of life lost due to premature mortality and years lived with disability, with years lived with disability calculated as the product of prevalence and a disability weight for each health state. These metrics are accompanied by their respective 95% uncertainty intervals (UIs), which provide a measure of statistical confidence. Ethical approval for this study was unnecessary as it relied solely on de-identified, aggregated data from the GBD study. The GBD study itself is approved by the University of Washington Institutional Review Board, which waives individual consent for such data.

All this data are accessible for free access through the Global Health Data Exchange (https://ghdx.healthdata.org/gbd-2021/sources),^[7]^ with detailed information on the data, methodologies, and statistical modeling available in previous reports.^[8]^ The GBD 2021 study applied advanced statistical models, including meta-regression-Bayesian regularized trimmed, and DisMod-MR 2.1, to harmonize data collected worldwide and reduce heterogeneity. All estimates, including those for pediatric subgroups, underwent internal validation and quality control within the standardized GBD framework, involving consistency checks and expert review by the Institute for Health Metrics and Evaluation.

This study focused specifically on urticaria in individuals under 20 years of age, who were categorized as children and adolescents. This classification is consistent with the widely accepted definitions of the World Health Organization, which defines adolescents as individuals aged 10 to 19 years and children as individuals under 10 years of age.^[9]^

### 2.2. Calculation of estimated annual percentage change (EAPC)

The EAPC is computed by applying a linear regression model to the natural logarithm of age-standardized rates (ASRs) across a defined time period.^[10,11]^ First, the ASRs are log-transformed, and then a linear regression is performed with time (in years) as the independent variable and the log-transformed ASRs as the dependent variable. The slope (β) obtained from this regression represents the EAPC, which reflects the average annual percentage change in the ASRs. The formula used for calculation is EAPC = 100 × (exp(β) − 1), where β is the slope, and exp(β) refers to the exponentiation of β.^[12]^

### 2.3. Sociodemographic index

The SDI is a composite indicator that measures a region’s socioeconomic status based on income, education, and fertility rate.^[13]^ For this study, countries and regions were categorized into quintiles based on their SDI values: low (SDI <20th percentile), low-middle (20–39th), middle (40–59th), high-middle (60–79th), and high (SDI ≥80th percentile).^[14]^

### 2.4. Disease definition

Urticaria, defined by GBD 2021, is a common skin condition characterized by red, itchy bumps that last from hours to days. These bumps can be caused by allergic reactions, infections, medications, or other unknown causes. The GBD project categorizes urticaria as 1 of the diseases of the skin and subcutaneous tissue. The international classification of diseases, 10th edition (ICD-10) code for urticaria is L50.^[15]^

### 2.5. Statistical analysis

The burden of urticaria was assessed by prevalence, incidence, DALYs, and ASR per 100,000 population. Decomposition analysis was used to quantify the contribution of population growth, aging, and epidemiologic changes to observed trends in prevalence. Projections to 2050 were made using a BAPC model with integrated nested Laplace approximations for accurate forecasting.^[16,17]^ All statistical analyses were conducted using R software (version 4.3.2; R Foundation for Statistical Computing, Vienna, Austria). Specifically, we used the “BAPC” and “integrated nested Laplace approximation” packages for Bayesian modeling, and the “ggplot2,” “dplyr,” and “Epi” packages for data visualization and epidemiological calculations. A 2-sided *P*-value < .05 was considered statistically significant.

## 3. Result

### 3.1. Global level

From 1990 to 2021, the prevalence, incidence, and DALYs of urticaria in children and adolescents increased globally (Table 1). By 2021, the estimated number of prevalent cases of urticaria in children and adolescents reached 31,225, 865.98 (95% UI: 25,693, 651.58, 38,651, 325.76), a 15.94% increase from 26,933, 159.42 cases (95% UI: 22,207, 685.32, 33,284, 588.95) in 1990. The global age-standardized prevalence rate (ASPR) of urticaria in children and adolescents rose from 1192.03 per 100,000 in 1990 to 1203.16 per 100,000 in 2021. Meanwhile, the number of incident cases surged by 15.01%, from 48,188,479.19 in 1990 to 55,421,308.75 in 2021. The number of DALYs showed a parallel trend, increasing from 1644,818.03 in 1990 to 1913,779.77 in 2021, a rise of 16.35%.

**Table 1:** Age-standardized prevalence, incidence, DALYs, and EAPC of urticaria in children and adolescents aged <20 years at the global and regional levels, 1990 to 2021.

	1990		2021		EAPC (1990–2021)
	Cases in 1990	ASR in 1990 (per 1 00 000 population)	Cases in 2021	ASR in 2021 (per 1 00 000 population)	
Prevalence (95% UI)					
Global	4,713,373.42 (3,294,180.67–6,768,623.82)	300.73 (190.71–458.56)	6,741,277.21 (4,636,005.85–9,778,157.52)	354.72 (223.18–548.92)	0.57 (0.54–0.60)
Sex					
Male	11,488,262.14 (14,243,649.34, 9,475,780.73)	990.93 (890.79, 1091.07)	13,393,895.11 (16,655,127.49, 10,996,504.95)	1001.05 (897.09, 1105.01)	0.0403 (0.0322, 0.0484)
Female	15,444,897.28 (19,089,309.10, 12,755,018.90)	1403.89 (1266.85, 1540.93)	17,831,970.87 (22,082,793.09, 14,676,056.31)	1418.37 (1274.63, 1562.11)	0.0342 (0.0245, 0.0440)
Age					
<5	10,238,963.37 (12,685,301.49, 8,387,804.72)	1651.61 (1584.16, 1719.06)	11,111,763.68 (13,775,792.74, 9,090,005.14)	1688.28 (1619.33, 1757.23)	0.0708 (0.0584, 0.0833)
5–9	7,558,887.77 (10,898,368.59, 5,359,302.20)	1295.37 (1242.47, 1348.27)	8,993,672.04 (12,949,397.15, 6,372,430.16)	1309.02 (1255.56, 1362.48)	0.0567 (0.0472, 0.0661)
10–14	4,936,326.85 (6,945,522.00, 3,381,579.17)	921.50 (883.86, 959.14)	6,159,715.75 (8,671,502.91, 4,212,396.89)	924.00 (886.26, 961.74)	0.0015 (−0.0013, 0.0042)
15–19	4,198,981.44 (5,860,423.29, 2,979,231.83)	808.39 (775.37, 841.41)	4,960,714.51 (6,914,926.38, 3,521,263.29)	795.01 (762.54, 827.48)	−0.0575 (−0.0692, −0.0457)
Socio-demographic inde					
High SDI	2,564,006.10 (3,015,615.76, 2,213,026.56)	1047.98 (925.13, 1170.83)	2,336,669.84 (2,703,953.78, 2,049,242.47)	1042.41 (919.72, 1165.10)	−0.0428 (−0.0526, −0.0330)
High-middle SDI	4,188,961.59 (5,217,542.31, 3,454,373.72)	1154.35 (1043.77, 1264.93)	3,332,072.37 (4,196,307.83, 2,719,905.82)	1122.67 (1017.83, 1227.51)	-0.0694 (−0.0913, −0.0475)
Middle SDI	8,509,034.00 (10,641,085.24, 7,005,554.71)	1121.51 (1034.89, 1208.13)	8,360,187.45 (10,527,375.42, 6,853,816.34)	1136.90 (1042.05, 1231.75)	0.0509 (0.0419, 0.0600)
Low-middle SDI	7,997,293.75 (9,969,043.21, 6,570,935.75)	1324.99 (1180.49, 1469.49)	9,929,650.35 (12,339,455.36, 8,114,107.97)	1323.74 (1178.64, 1468.84)	−0.0020 (−0.0044, 0.0005)
Low SDI	3,649,278.07 (4,526,005.13, 3,000,579.24)	1247.10 (1106.82, 1387.38)	7,244,053.55 (9,039,161.32, 5,940,570.82)	1227.15 (1089.65, 1364.65)	−0.0504 (−0.0513, −0.0496)
Region					
Andean Latin America	217,264.29 (274,744.02, 177,715.15)	1139.37 (1009.47, 1269.27)	266,488.7 (336,791.87, 217,427.09)	1138.57 (1007.45, 1269.69)	−0.0076 (−0.0099, −0.0053)
Australasia	68,361.68 (84,197.27, 56,046.78)	1115.05 (1004.98, 1225.12)	82,302.82 (101,882.75, 67,310.2)	1115.22 (1004.54, 1225.90)	−0.0036 (−0.0066, −0.0005)
Caribbean	171,012.36 (214,894.66, 140,234.54)	1140.58 (1010.43, 1270.73)	170,724.86 (215,425.8,139,390.19)	1140.53 (1009.65, 1271.41)	−0.0036 (−0.0050, −0.0022)
Central Asia	547,453.78 (683,013.3, 450,449.04)	1682.18 (1443.25, 1921.11)	596,332.45 (747,023.67, 489,542.33)	1679.83 (1440.76, 1918.90)	−0.0162 (−0.0214, −0.0110)
Central Europe	682,770.35 (836,727.95, 571,609.96)	1828.20 (1550.66, 2105.74)	409,208.4 (489,315.16, 347,082.6)	1820.94 (1542.53, 2099.35)	−0.0075 (−0.0180, 0.0029)
Central Latin America	971,734.93 (1,210,685.34, 797,476.26)	1172.00 (1041.42, 1302.58)	967,499.93 (1,206,401.78, 790,095.53)	1172.90 (1040.82, 1304.98)	−0.0003 (−0.0030, 0.0025)
Central Sub-Saharan Africa	370,250.38 (465,521.75, 302,964.98)	1136.72 (1007.89, 1265.55)	853,749.8 (1,080,005.57, 698,214.59)	1140.89 (1010.23, 1271.55)	0.0128 (0.0111, 0.0144)
East Asia	4,470,322.45 (5,556,226.05, 3,657,609.67)	978.95 (933.42, 1024.48)	3,356,827.3 (4,255,511.71, 2,715,176.93)	980.17 (931.28, 1029.06)	−0.0160 (−0.0264, −0.0055)
Eastern Europe	1,165,132.01 (1,450,570.25, 954,837.04)	1757.12 (1514.47, 1999.77)	778,659.05 (978,998.47, 632,534.43)	1756.31 (1514.02, 1998.60)	−0.0073 (−0.0184, 0.0037)
Eastern Sub-Saharan Africa	1,342,338.3 (1,671,320.62, 1,103,205.44)	1159.51 (1030.48, 1288.54)	2,663,016.08 (3,337,776.12, 2,178,583.99)	1161.67 (1031.07, 1292.27)	0.0058 (0.0044, 0.0073)
High-income Asia Pacific	501,617.28 (630,638.89, 408,609.97)	1067.24 (947.59, 1186.89)	311,947.38 (390,767.24, 253,077.45)	1069.56 (949.19, 1189.93)	0.0092 (0.0046, 0.0138)
High-income North America	1,006,776.98 (1,128,124.41, 908,521.59)	1244.94 (1079.52, 1410.36)	1,094,254.05 (1,211,093.68, 1,000,014.94)	1283.92 (1112.09, 1455.75)	0.0262 (0.0003, 0.0521)
North Africa and Middle East	2,277,787.42 (2,799,266.58, 1,876,228.61)	1265.13 (1118.81, 1411.45)	2,992,262.61 (3,683,774.98, 2,455,361.11)	1275.11 (1126.36, 1423.86)	0.0243 (0.0167, 0.0320)
Oceania	32,543.79 (40,980.77, 26,412.12)	958.10 (901.31, 1014.89)	61,754.48 (77,699.15, 50,158.42)	957.59 (900.68, 1014.50)	−0.0033 (−0.0051, −0.0016)
South Asia	7,827,390.91 (9,747,007.02, 6,434,906.96)	1411.70 (1243.77, 1579.63)	9,358,028.24 (11,680,474.54, 7,681,756.91)	1425.23 (1254.83, 1595.63)	0.0301 (0.0242, 0.0361)
Southeast Asia	2,173,114.1 (2,730,546.8 3,1,742,743.17)	987.12 (963.19, 1011.05)	2,274,566.74 (2,857,697.31, 1,819,931.23)	989.78 (965.89, 1013.67)	0.0089 (0.0080, 0.0098)
Southern Latin America	198,610.08 (248,588.94, 162,469.97)	1031.11 (912.68, 1149.54)	192,987.67 (242,707.76, 156,900.67)	1032.57 (912.71, 1152.43)	−0.0001 (−0.0045, 0.0044)
Southern Sub-Saharan Africa	315,539.09 (392,276.11, 258,865.42)	1183.12 (1052.15, 1314.09)	365,163.03 (455,106.75, 298,758.07)	1181.02 (1049.11, 1312.93)	−0.0088 (−0.0111, −0.0066)
Tropical Latin America	821,248.09 (1,025,660.58, 666,793.7)	1202.03 (1069.38, 1334.68)	785,181.93 (974,692.29, 643,163.77)	1198.24 (1065.97, 1330.51)	−0.0100 (−0.0125, −0.0075)
Western Europe	456,754.7 (552,423.92, 381,175.11)	471.91 (430.79, 513.03)	430,434.95 (523,175.31, 359,043.84)	478.84 (437.97, 519.71)	0.0461 (0.0441, 0.0482)
Western Sub-Saharan Africa	1,315,136.44 (1,632,571.24, 1,078,944.08)	1166.86 (1038.15, 1295.57)	3,214,475.52 (4,012,803.61, 2,627,940.38)	1169.74 (1039.56, 1299.92)	0.0126 (0.0075, 0.0178)
Incidence (95% UI)					
Global	48,188,479.19 (59,253,291.28, 39,698,524.90)	2131.28 (1886.04, 2376.52)	55,421,308.75 (68,294,618.59, 45,555,550.93)	2142.29 (1889.88, 2394.7)	0.0239 (0.0127, 0.0352)
Sex					
Male	20,544,959.48 (25,337,816.84, 16,971,893.72)	1770.51 (1562.61, 1978.41)	23,769,118.49 (29,384,054.62, 19,602,899.45)	1782.16 (1569.08, 1995.24)	0.0316 (0.0200, 0.0433)
Female	27,643,519.71 (34,017,255.41, 22,822,023.90)	2511.49 (2225.84, 2797.14)	31,652,190.26 (38,916,299.50, 25,914,330.41, 441,723)	2525.81 (2231.14, 2820.48)	0.0220 (0.0093, 0.0348)
Age					
<5	19,951,795.03 (24,702,945.90, 16,193,887.83)	3218.35 (3086.91, 3349.79)	21,469,908.94 (26,619,297.99, 17,402,777.31)	3262.05 (3128.82, 3395.28)	0.0533 (0.0333, 0.0734)
5–9	12,556,066.31 (18,130,151.14, 8,708,487.28)	2151.74 (2063.86, 2239.62)	14,917,945.21 (21,551,244.08, 10,325,413.82)	2171.29 (2082.61, 2259.97)	0.0495 (0.0412, 0.0579)
10–14	8,394,043.94 (11,850,184.39, 5,742,852.40)	1566.98 (1502.98, 1630.98)	10,443,185.03 (14,710,810.74, 7,184,658.95)	1566.55 (1502.57, 1630.53)	−0.0109 (−0.0143, −0.0074)
15–19	7,286,573.92 (10,058,311.00, 5,036,793.49)	1402.82 (1345.53, 1460.11)	8,590,269.57 (11,887,598.51, 5,984,300.44)	1376.69 (1320.46, 1432.92)	−0.0629 (−0.0751, −0.0508)
Socio-demographic inde					
High SDI	4,548,452.82 (5,327,162.29, 3,904,669.59)	1863.49 (1616.66, 2110.32)	4,130,811.71 (4,760,032.68, 3,599,057.69)	1850.67 (1606.24, 2095.10)	−0.0404 (−0.0515, −0.0294)
High-middle SDI	7,453,459.07 (9,189,391.87, 6,112,692.42)	2057.61 (1830.80, 2284.42)	5,881,847.60 (7,339,069.21, 4,768,033.05)	1992.82 (1781.03, 2204.61)	−0.0644 (−0.0827, −0.0461)
Middle SDI	15,226,777.92 (18,877,193.17, 12,429,785.93)	2008.71 (1823.77, 2193.65)	14,824,548.61 (18,468,114.92, 12,010,365.58)	2025.99 (1828.88, 2223.10)	0.0428 (0.0303, 0.0553)
Low-middle SDI	14,327,911.30 (17,882,891.86, 11,656,531.97)	2366.84 (2067.52, 2666.16)	17,609,099.51 (21,835,431.53, 14,432,348.43)	2354.37 (2058.23, 2650.51)	−0.0151 (−0.0167, −0.0135)
Low SDI	6,588,204.68 (8,189,498.82, 5,381,954.45)	2232.71 (1939.92, 2525.50)	12,933,720.21 (16,028,497.85, 10,578,673.99)	2187.11 (1904.40, 2469.82)	−0.0693 (−0.0706, −0.0680)
Region					
Andean Latin America	388,182.33 (483,247.64, 316,670.77)	2033.42 (1765.82, 2301.02)	473,037.87 (588,176.52, 385,530.85)	2024.18 (1757.81, 2290.55)	−0.0139 (−0.0148, −0.0130)
Australasia	121,754.54 (147,302.03, 99,364.03)	1990.11 (1762.74, 2217.48)	145,994.56 (177,947.19, 118,697.06)	1985.98 (1759.55, 2212.41)	−0.0023 (−0.0053, 0.0006)
Caribbean	305,509.04 (377,592.68, 250,582.56)	2034.46 (1766.87, 2302.05)	302,970.97 (375,520.29, 247,490.61)	2028.62 (1762.20, 2295.04)	−0.0070 (−0.0082, −0.0057)
Central Asia	973,447.24 (1,206,177.44, 794,748.28)	2975.94 (2498.42, 3453.46)	1,057,180.21 (1,314,794.86, 859,525.07)	2972.05 (2494.07, 3450.03)	0.0069 (0.0011, 0.0126)
Central Europe	1,196,123.77 (1,451,369.02, 992,738.17)	3220.39 (2676.59, 3764.19)	717,340.86 (853,729.31, 608,715.85)	3206.39 (2661.28, 3751.50)	0.0062 (−0.0019, 0.0144)
Central Latin America	1,735,868.92 (2,162,163.01, 1,414,056.06)	2090.67 (1821.58, 2359.76)	1,708,830.75 (2,129,069.99, 1,389,092.68)	2082.32 (1814.82, 2349.82)	−0.0104 (−0.0125, −0.0083)
Central Sub-Saharan Africa	671,403.02 (833,090.16, 547,900.37)	2039.06 (1769.07, 2309.05)	1,522,704.34 (1,907,118.18, 1,240,608.54)	2031.47 (1764.48, 2298.46)	−0.0124 (−0.0141, −0.0107)
East Asia	8,042,045.9 (9,860,230.86, 6,519,442.11)	1764.28 (1658.08, 1870.48)	5,958,696.04 (7,454,878.36, 4,767,701.52)	1747.17 (1643.23, 1851.11)	−0.0246 (−0.0278, −0.0214)
Eastern Europe	2,042,527.65 (2,551,789.29, 1,653,413.67)	3087.92 (2611.49, 3564.35)	1,355,454.24 (1,703,187.44, 1,095,424.73)	3088.28 (2611.70, 3564.86)	0.0102 (0.0008, 0.0196)
Eastern Sub-Saharan Africa	2,430,082.12 (3,013,919.75, 1,977,838.92)	2080.11 (1809.21, 2351.01)	4,752,944.03 (5,905,654.48, 3,871,759.13)	2070.49 (1802.33, 2338.65)	−0.0186 (−0.0208, −0.0165)
High-income Asia Pacific	883,295.75 (1,094,488.89, 712,209.34)	1894.93 (1653.50, 2136.36)	548,284.94 (682,854.75, 442,294.23)	1895.48 (1654.35, 2136.61)	0.0012 (−0.0031, 0.0054)
High-income North America	1,788,143.51 (1,980,358.89, 1,604,954.15)	2211.31 (1880.45, 2542.17)	1,931,891.93 (2,123,639.36, 1,745,290.25)	2276.91 (1936.57, 2617.25)	0.0269 (0.0014, 0.0523)
North Africa and Middle East	4,062,416.79 (5,043,152.58, 3,325,933.09)	2251.30 (1953.21, 2549.39)	5,288,793.81 (6,580,899.24, 4,337,926.92)	2258.98 (1960.21, 2557.75)	0.0164 (0.0138, 0.0190)
Oceania	58,810.58 (73,286.98, 47,568.88)	1724.37 (1596.75, 1851.99)	111,668.33 (139,076.75, 90,424.72)	1723.23 (1595.55, 1850.91)	−0.0019 (−0.0024, −0.0014)
South Asia	13,989,029.12 (17,414,246.36, 11,383,591.81)	2517.96 (2174.11, 2861.81)	16,540,321.95 (20,455,368.46, 13,462,483.95)	2533.10 (2188.68, 2877.52)	0.0165 (0.0112, 0.0218)
Southeast Asia	3,924,537.3 (4,885,637.96, 3,126,665.1)	1784.29 (1733.67, 1834.91)	4,091,658.58 (5,068,321.91, 3,250,085.17)	1787.32 (1736.49, 1838.15)	0.0069 (0.0064, 0.0074)
Southern Latin America	352,958.06 (436,459.56, 287,337.75)	1833.62 (1593.66, 2073.58)	338,565.63 (420,653.35, 274,102.52)	1824.07 (1586.89, 2061.25)	−0.0064 (−0.0117, −0.0011)
Southern Sub-Saharan Africa	563,624.94 (703,697.82, 458,957.22)	2109.41 (1839.92, 2378.90)	647,794.51 (809,963.67, 525,252.37)	2100.22 (1831.44, 2369.00)	−0.0142 (−0.0165, −0.0118)
Tropical Latin America	1,451,363.42 (1,815,474.83, 1,172,862.13)	2134.14 (1865.11, 2403.17)	1,394,890.81 (1,740,977.68, 1,132,302.54)	2131.66 (1861.66, 2401.66)	−0.0036 (−0.0047, −0.0024)
Western Europe	822,144.54 (993,086.16, 679,196.84)	849.38 (766.14, 932.62)	771,841.8 (932,710.61, 637,383.19)	859.63 (777.57, 941.69)	0.0416 (0.0366, 0.0466)
Western Sub-Saharan Africa	2,385,210.66 (2,958,104.41, 1,941,428.1)	2094.26 (1823.22, 2365.30)	5,760,442.59 (7,167,612.31, 4,692,177.95)	2087.95 (1819.07, 2356.83)	−0.0074 (−0.0100, −0.0048)
DALYs (95% UI)					
Global	1,644,818.03 (2,390,025.34, 1,055,481.89)	72.8 (65.59, 80.01)	1,913,779.77 (2,787,576.11, 1,222,777.34)	73.75 (66.13, 81.37)	0.0482 (0.0409, 0.0555)
Sex					
Male	701,897.31 (1,020,793.16, 446,615.93)	60.54 (54.44, 66.64)	822,272.97 (1,203,109.68, 520,369.85)	61.46 (55.06, 67.86)	0.0569 (0.0496, 0.0642)
Female	942,920.72 (1,371,306.62, 609,067.29)	85.71 (77.28, 94.14)	1,091,506.80 (1,588,953.73, 704,215.74)	86.84 (77.91, 95.77)	0.0457 (0.0365, 0.0549)
Age					
<5	625,361.73 (922,058.69, 401,362.91)	100.87 (96.75, 104.99)	683,146.66 (1,012,423.13, 436,664.84)	103.79 (99.55, 108.03)	0.0939 (0.0833, 0.1045)
5–9	462,785.65 (730,071.13, 265,635.74)	79.31 (76.07, 82.55)	552,224.99 (872,743.49, 315,663.38)	80.38 (77.10, 83.66)	0.0666 (0.0577, 0.0755)
10–14	301,046.72 (471,359.51, 173,953.20)	56.20 (53.90, 58.50)	376,441.80 (589,363.15, 218,758.04)	56.47 (54.16, 58.78)	0.0080 (0.0050, 0.0110)
15–19	255,623.93 (414,556.70, 149,979.28)	49.21 (47.20, 51.22)	301,966.31 (491,624.54, 177,893.34)	48.39 (46.41, 50.37)	−0.0525 (−0.0657, −0.0392)
Socio-demographic inde					
High SDI	157,868.47 (227,449.70, 102,988.41)	64.56 (56.89, 72.23)	143,789.53 (207,921.03, 93,799.09)	64.21 (56.51, 71.91)	−0.0425 (−0.0528, −0.0323)
High-middle SDI	257,782.26 (376,029.83, 165,486.50)	71.05 (64.18, 77.92)	205,505.38 (300,369.91, 131,748.45)	69.27 (62.71, 75.83)	−0.0606 (−0.0823, −0.0388)
Middle SDI	521,159.71 (762,092.56, 333,061.92)	68.70 (63.36, 74.04)	513,811.27 (753,073.51, 326,496.56)	69.89 (63.98, 75.80)	0.0635 (0.0545, 0.0724)
Low-middle SDI	485,697.73 (710,911.00, 310,124.68)	80.47 (71.68, 89.26)	607,473.76 (885,426.96, 387,459.83)	81.00 (72.05, 89.95)	0.0240 (0.0213, 0.0266)
Low SDI	220,801.92 (321,911.65, 141,579.60)	75.46 (66.97, 83.95)	441,774.10 (647,323.53, 282,964.09)	74.83 (66.39, 83.27)	−0.0200 (−0.0223, −0.0178)
Region					
Andean Latin America	13,286.76 (19,744.75, 8399.2)	69.68 (61.74, 77.62)	48,043.03 (71,226.87, 30,468.87)	70.05 (61.91, 78.19)	0.0169 (0.0130, 0.0207)
Australasia	4198.87 (6154.35, 2682.51)	68.54 (61.63, 75.45)	5057.71 (7405.38, 3214.77)	68.58 (61.60, 75.56)	0.0030 (−0.0011, 0.0070)
Caribbean	10,454.31 (15,391.75, 6670.51)	66.96 (61.17, 72.75)	571,178.59 (843,879.76, 364,874.84)	69.71 (61.62, 77.80)	0.0540 (−0.0256, 0.1336)
Central Asia	33,527.55 (49,335.62, 21,730.77)	103.02 (88.36, 117.68)	26,411.54 (38,980,16,835.97)	103.28 (88.52, 118.04)	−0.0019 (−0.0074, 0.0036)
Central Europe	42,066.36 (61,035.15, 27,371.84)	112.68 (95.46, 129.90)	25,236.74 (36,536.16, 16,507.76)	112.35 (95.03, 129.67)	−0.0011 (−0.0104, 0.0081)
Central Latin America	59,636.55 (87,226.87, 38,367.69)	71.92 (63.83, 80.01)	36,668.01 (53,257.3,23,528.95)	72.30 (64.06, 80.54)	0.0113 (0.0084, 0.0142)
Central Sub-Saharan Africa	22,454.01 (32,644.51, 14,467.67)	68.91 (61.03, 76.79)	10,431.69 (15,317.6,6669.41)	69.77 (61.69, 77.85)	0.0490 (0.0418, 0.0563)
East Asia	275,383.89 (408,468.9,172,101.08)	60.32 (57.46, 63.18)	67,293.07 (96,047.55, 45,006.02)	60.63 (57.53, 63.73)	−0.0038 (−0.0135, 0.0059)
Eastern Europe	71,769.92 (105,701.7,46,148.18)	108.25 (93.20, 123.30)	59,605.07 (86,980.66, 37,840.35)	108.40 (93.33, 123.47)	0.0015 (−0.0098, 0.0129)
Eastern Sub-Saharan Africa	81,404.56 (119,295.33, 51,945.07)	70.32 (62.49, 78.15)	16,392.95 (24,145.75, 10,308.56)	71.03 (62.96, 79.10)	0.0433 (0.0404, 0.0462)
High-income Asia Pacific	30,877.87 (45,381.84, 19,669.16)	65.76 (58.30, 73.22)	19,227.62 (28,564.99, 12,172.36)	65.98 (58.46, 73.50)	0.0117 (0.0075, 0.0159)
High-income North America	62,032.38 (88,401.39, 41,467.07)	76.73 (66.39, 87.07)	48,084.4 (70,266.66, 30,538.03)	79.06 (68.28, 89.84)	0.0239 (−0.0027, 0.0504)
North Africa and Middle East	139,472.39 (202,007.36, 89,910.87)	77.45 (68.42, 86.48)	11,872.64 (17,416.22, 7477.29)	78.34 (69.07, 87.61)	0.0378 (0.0293, 0.0462)
Oceania	1987.78 (2966.23, 1259.17)	58.51 (55.01, 62.01)	207,593.02 (303,940.98, 129,997.5)	58.70 (55.16, 62.24)	0.0108 (0.0064, 0.0152)
South Asia	474,069.36 (697,229.25, 302,682.54)	85.50 (75.34, 95.66)	3786.17 (5681.27, 2383.9)	87.01 (76.56, 97.46)	0.0557 (0.0501, 0.0613)
Southeast Asia	132,902.22 (197,157.06, 83,876.2)	60.37 (58.87, 61.87)	139,749.54 (209,594.98, 88,515.36)	60.83 (59.32, 62.34)	0.0287 (0.0266, 0.0307)
Southern Latin America	12,208.47 (17,971.44, 7798.12)	63.39 (56.04, 70.74)	52,223.82 (76,798.43, 33,154.87)	63.56 (56.10, 71.02)	−0.0011 (−0.0080, 0.0058)
Southern Sub-Saharan Africa	19,281.53 (28,484.16, 12,400.2)	72.30 (64.28, 80.32)	162,843.35 (238,770.85, 103,584.57)	72.35 (64.18, 80.52)	0.0058 (0.0023, 0.0092)
Tropical Latin America	50,117.82 (74,365.85, 32,022.75)	73.36 (65.18, 81.54)	195,897.25 (288,073.18, 124,151.68)	73.40 (65.17, 81.63)	0.0056 (0.0028, 0.0085)
Western Europe	28,057.3 (41,651.76, 17,797.28)	29.02 (26.44, 31.60)	22,368.07 (33,230.43, 14,189.32)	29.42 (26.84, 32.00)	0.0464 (0.0427, 0.0500)
Western Sub-Saharan Africa	79,628.13 (116,526.11, 50,866.87)	70.64 (62.83, 78.45)	183,815.48 (267,266.73, 119,542.51)	71.27 (63.29, 79.25)	0.0385 (0.0294, 0.0475)

ASR = age-standardized rate, DALYs = disability-adjusted life-years, EAPC = estimated annual percentage change, SDI = sociodemographic index, UI = uncertainty interval.

**Figure 1.**
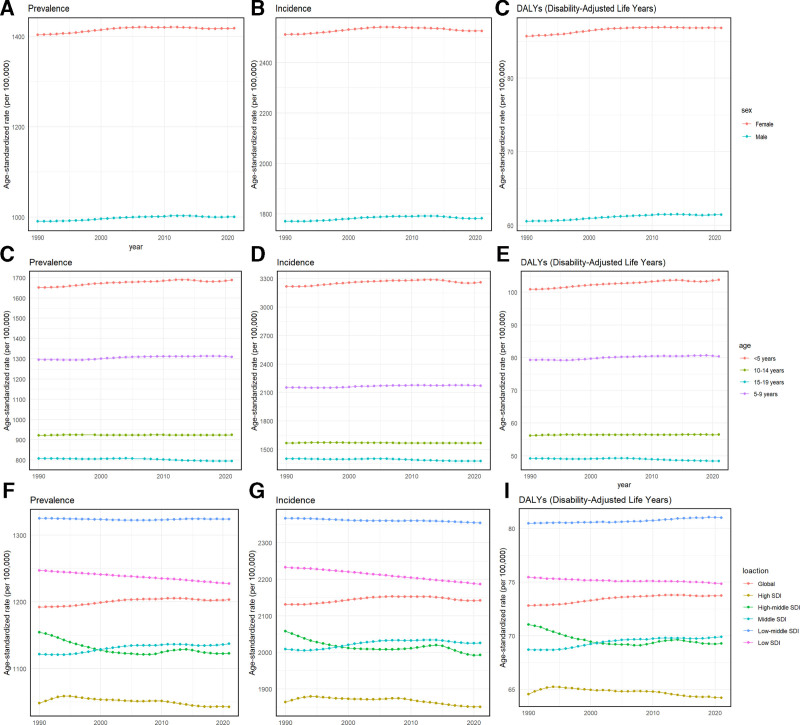
Global trends by gender for age-standardized prevalence rate. (A) incidence rate (B) and disability-adjusted life-year (DALY) rate (C) of urticaria in children and adolescents aged <20 years from 1990 to 2021. Global trends by age groups for age-standardized prevalence rate (D), incidence rate (E) and DALY rate (F) of urticaria from 1990 to 2021. Global trends by SDI quintiles for age-standardized prevalence rate (G), incidence rate (H) and DALY rate (I) of urticaria from 1990 to 2021. DALY = disability-adjusted life-year, SDI = sociodemographic index.

From 1990 to 2021, the global ASPR, ASIR, and age-standardized DALY rate of urticaria in children and adolescents showed a slight upward trend (Fig. 1G–I). Specifically, the ASPR was 0.0346 (95% UI: 0.0266, 0.0426), the ASIR was 0.0239 (95% UI: 0.0127, 0.0352), and the age-standardized DALY rate was 0.0482 (95% UI: 0.0409, 0.0555) (Table 1). Although females consistently had higher ASPR, ASIR, and DALY rates, the annual increase in these indicators for males was slightly higher than for females from 1990 to 2021 (Fig. 1A–C). This comprehensive analysis highlights the escalating impact of urticaria on children and adolescents, affecting both sexes across different regions globally.

### 3.2. SDI regional level

From 1990 to 2021, low-middle and middle SDI regions had higher ASPR, ASIR, and DALYs compared to other regions, with the lowest rates in high SDI regions. In 2021, the ASPR in low-middle SDI regions was 1323.74 (95% UI: 1178.64, 1468.84), the ASIR was 2354.37 (95% UI: 2058.23, 2650.51), and the age-standardized DALY rate was 81.00 (95% UI: 72.05, 89.95). The EAPC for these indicators showed a negative trend in most SDI regions except for middle SDI regions, where the EAPC was 0.0635 (0.0545, 0.0724) (Table 1; Fig. 1G–I).

**Figure 2.**
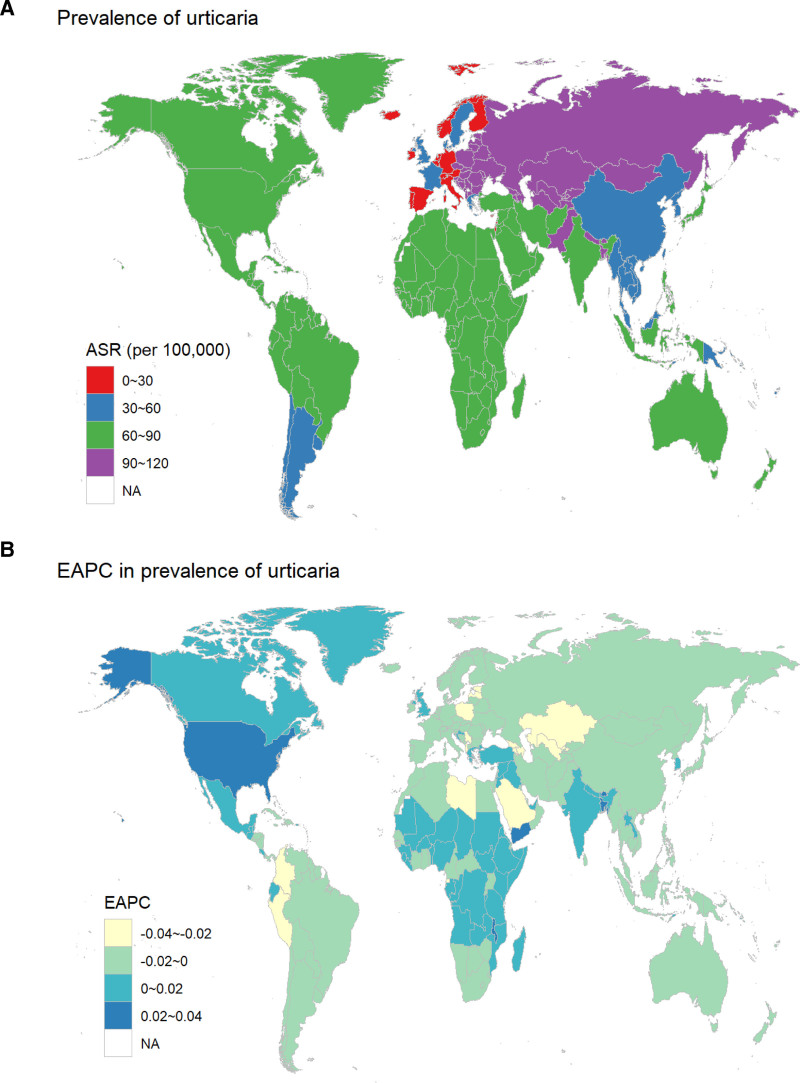
The global prevalence of urticaria in children and adolescents aged <20 years in 204 countries and territories. (A) Age-standardized prevalence rate in 2021. (B) Estimated annual percentage change (EAPC) in prevalence from 1990 to 2021. EAPC = estimated annual percentage change.

We also examined the global correlation between SDI and urticaria rates. Prevalence (*r* = −0.1523, *P* < .001), incidence (*r* = −0.1807, *P *< .001), and DALY rates (*r* = −0.1397, *P* < .001) were negatively correlated with SDI. High SDI regions like high-income Asia Pacific and Western Europe had the lowest rates, while low SDI regions like sub-Saharan Africa had the highest prevalence rates (Fig. 2).

### 3.3. GBD regional level

South Asia emerged as a hotspot for urticaria among children and adolescents, reporting the highest burden of both prevalent and incident cases globally in 2021. Conversely, Oceania reported the lowest number of cases, representing a stark contrast in the geographic distribution of the disease (Table 1). The burden of urticaria displayed significant regional heterogeneity. A pronounced intra-European disparity was evident, with the highest (ASRs, ASPR, ASIR, and DALYs) clustered in Central and Eastern Europe, in contrast to the lowest rates found in Western Europe. Analysis of trends identified Western Europe and South Asia as regions with the most concerning increases in prevalence/incidence and DALYs, respectively, while several Asian regions showed improving trends. All regional estimates and EAPCs are presented in Table 1

### 3.4. Countries level

**Figure 3.**
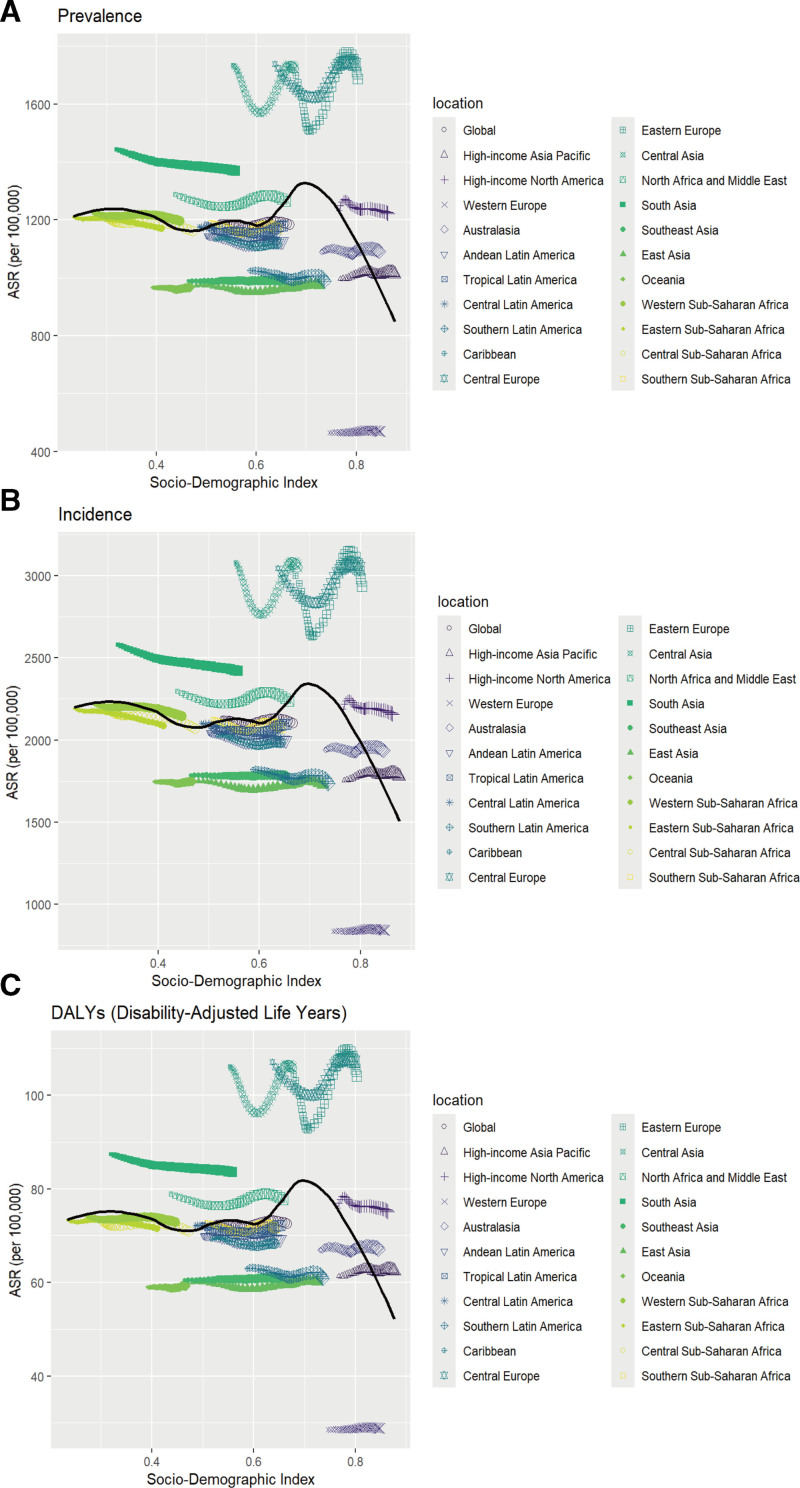
The associations between the sociodemographic index and urticaria across 21 GBD regions in children and adolescents aged <20 years. (A) Association between age-standardized urticaria prevalence rate and sociodemographic index. (B) Association between age-standardized urticaria incidence rate and sociodemographic index. (C) Association between age-standardized urticaria DALYs rate and sociodemographic index. ASR = age-standardized rate, DALYs = disability-adjusted life-years, EAPC = estimated annual percentage change, GBD = global burden of disease.

From 1990 to 2021, the burden of urticaria in the under-20 population exhibited substantial heterogeneity across the 204 countries analyzed. A clear pattern emerged, with Italy consistently demonstrating the lowest ASRs for prevalence, incidence, and DALYs in 2021. In stark contrast, Nepal carried the highest burden for both incidence and DALYs, while Ukraine recorded the highest prevalence rate (Table S1, Supplemental Digital Content, https://links.lww.com/MD/R296; Fig. 3A; Figures S1A and S2A, Supplemental Digital Content, https://links.lww.com/MD/R296). Regarding temporal trends, the United States of America was among the countries with the most significant increases in both prevalence and incidence over the 31-years period. Conversely, several nations, including Saudi Arabia and Equatorial Guinea, experienced notable declines. These findings suggest that the urticaria burden and its temporal evolution are not uniform and may be influenced by regional socioeconomic and healthcare factors (Table S1, Supplemental Digital Content, https://links.lww.com/MD/R296; Fig. 3B; Figures S1B and S2B, Supplemental Digital Content, https://links.lww.com/MD/R296).

### 3.5. Age and sex patterns

In 2021, the global situation of urticaria in children and adolescents revealed distinct patterns by age and sex. Figure 1D–F shows that prevalence, incidence, and DALY rates were consistently highest in children under 5 years old, declining with age. From 1990 to 2021, gender differences in ASPR, ASIR, and the age-standardized DALYs rate persisted, with females consistently reporting higher rates than males. However, the EAPC for prevalence, incidence, and DALY rates increased more significantly in males than in females (Fig. 1A and B; Table 1).

### 3.6. Decomposition analysis

**Figure 4.**
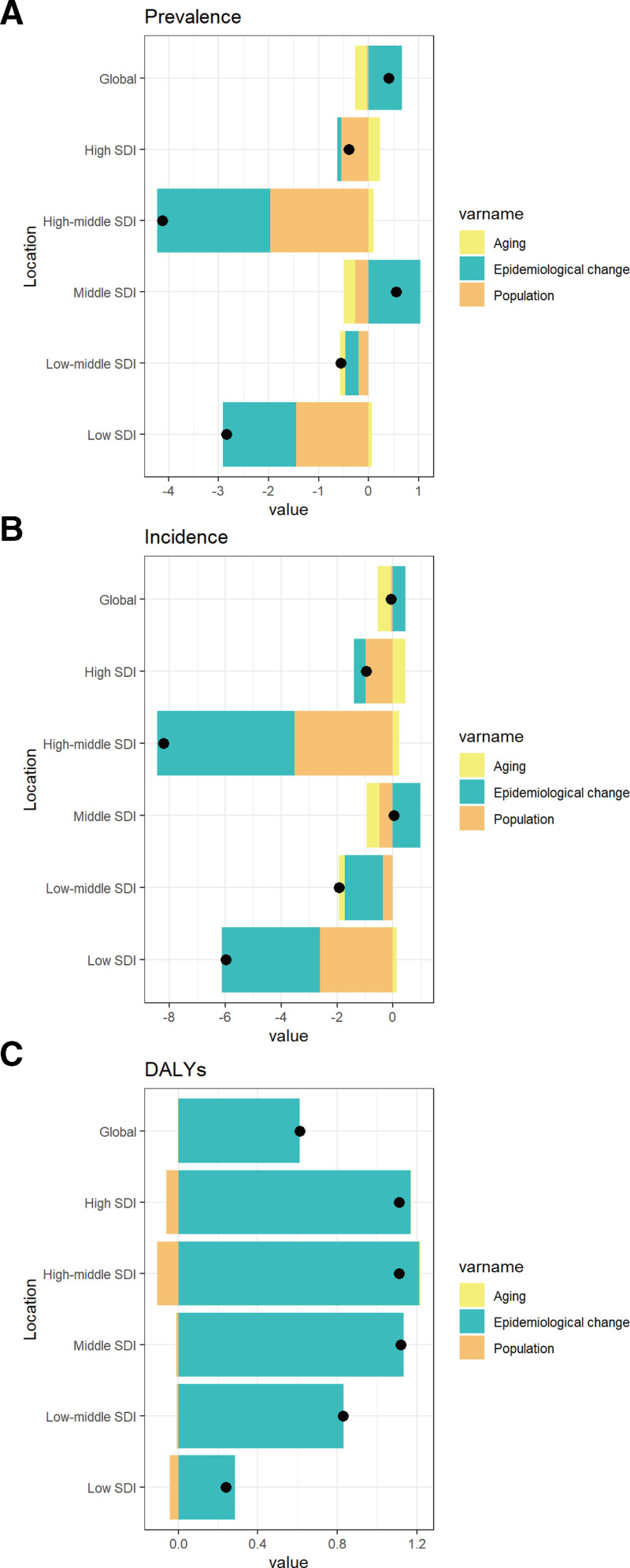
Decomposition analysis of the age-standardized prevalence rate. (A) incidence rate (B) and disability-adjusted life-year (DALY) rate (C) of urticaria in children and adolescents aged <20 years by SDI, 1990–2021. SDI = sociodemographic index.

Epidemiological changes positively impact urticaria incidence in middle, low-middle, and low SDI regions, while population changes have a negative effect. In high SDI regions, aging minimally affects prevalence. Both epidemiological and population changes significantly influence incidence, with epidemiological changes having a positive impact and population changes having a negative impact across all SDI regions. Globally and in all SDI regions, epidemiological changes are the main drivers of DALYs related to urticaria in children and adolescents, with population changes and aging having relatively minor effects (Fig. 4A–C).

### 3.7. Future burden of urticaria

**Figure 5.**
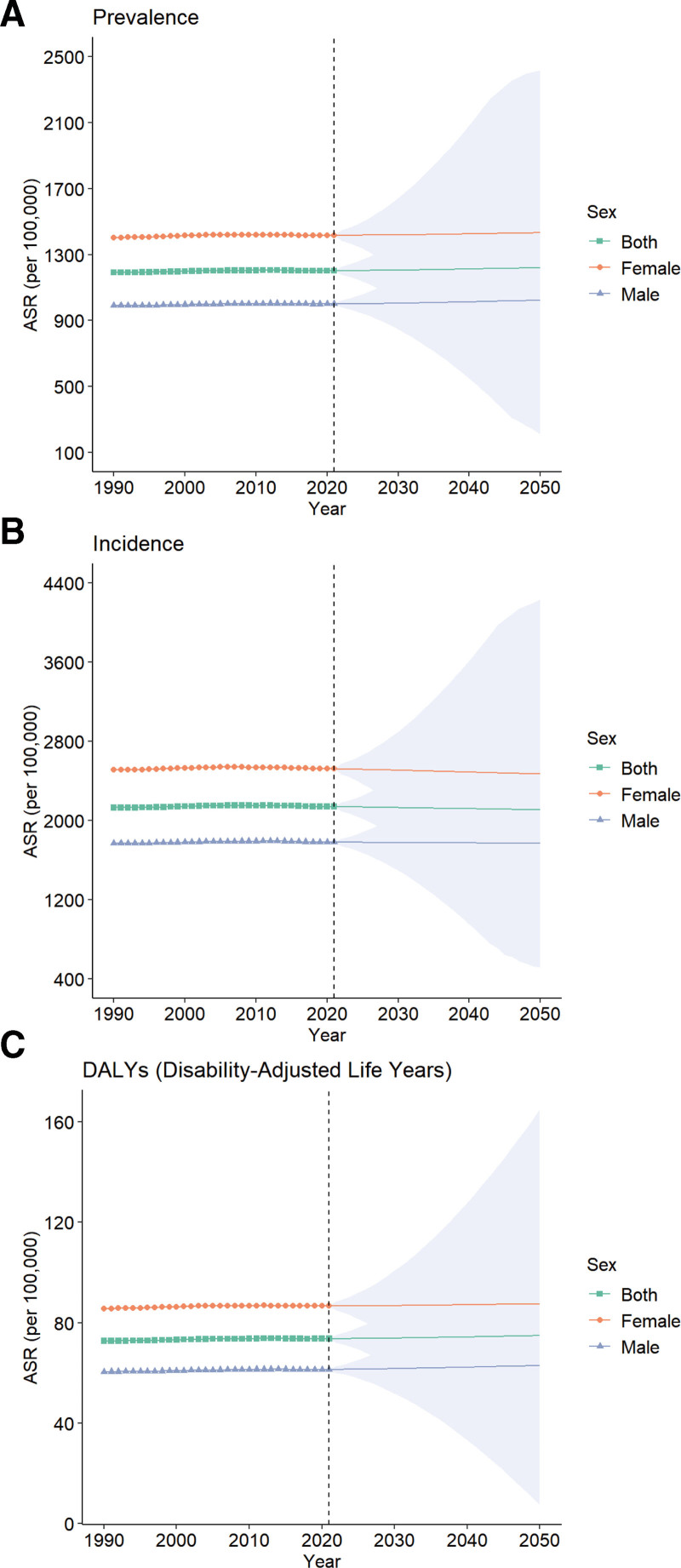
Projected trends in the age-standardized prevalence rate. (A) incidence rate (B) and disability-adjusted life-year (DALY) rate (C) of urticaria in children and adolescents aged <20 years from 2022 to 2050. ASR = age-standardized rate.

Using the BAPC model, we estimated trends in age-standardized incidence, prevalence, and DALY rates per 100,000 individuals from 1990 to 2021 and projected them to 2050. From 2021 to 2050, ASPR, ASIR, and DALY rates for urticaria among children and adolescents are expected to slightly increase globally (Fig. 5A–C). Similarly, the urticaria-related metrics for females have consistently been higher than those for males. The confidence intervals are widening, indicating increased uncertainty in long-term forecasts. The projected upward trend implies a gradual increase in the burden of urticaria by 2050, emphasizing the importance of sustained public health attention and preventive measures targeting children and adolescents.

## 4. Discussion

Using GBD 2021 data, this study provides the first global, regional, and national assessment of urticaria burden in individuals under 20. Unlike existing research that often extrapolates from adult data or relies on hospital-based studies,^[18]^ pediatric urticaria epidemiology varies significantly by region and age. Our findings reveal a rising global burden with notable disparities across age, gender, and SDI groups, highlighting the need for targeted interventions. Research on pediatric urticaria is essential for optimizing treatment, reducing disease burden, and improving children’s quality of life.

Our study demonstrates that from 1990 to 2021, the ASPR, ASIR, and DALY rates of urticaria among children and adolescents increased globally. Unlike most diseases, urticaria is characterized by recurrence within the same year, with each relapse counted as a new case: a key factor likely contributing to its rising incidence.^[19,20]^ Notably, acute urticaria in children is often self-limiting, whereas chronic CSU involves more complex etiologies, including air pollution,^[21]^ infections,^[22]^ drug allergens,^[23]^ and genetic factors.^[24]^ For example, data from the GBD study reveal that air pollution, including exposure to particulate matter and ozone, is significantly associated with an increased incidence of urticaria.^[25]^ The persistent growth in urticaria’s disease burden underscores the urgent need to investigate its underlying causes and risk factors.

Our findings confirm that urticaria is a prevalent skin disorder exhibiting significant regional and national disparities among children and adolescents, consistent with existing data.^[26]^ Multiple factors, including genetic predisposition, dietary habits, lifestyle, and socioeconomic conditions, have been reported to contribute to urticaria development.^[27,28]^ At the regional level, Central and Eastern Europe demonstrated the highest ASPR, incidence, and DALY rates for pediatric urticaria, while Western Europe and Oceania showed the lowest. This pattern may be linked to prolonged industrialization in Central/Eastern Europe, where heightened air pollution and chemical exposures could elevate disease risk.^[21,29]^

Nationally, Ukraine had the highest ASPR, whereas Nepal demonstrated peak ASPR and age-standardized DALY rates. Climatic factors contribute significantly. Ukraine’s temperate continental climate features extreme seasonal variations (harsh winters and hot summers), which are known triggers for urticaria and angioedema.^[30,31]^ Nepal’s diverse topography, from tropical monsoons to alpine zones, exposes populations to skin-irritating conditions that may exacerbate urticaria onset and persistence.^[32]^ Critically, Nepal’s healthcare resource limitations, particularly in remote mountainous regions,^[33]^ likely delay effective treatment, compounding disease burden. These findings highlight the interplay of environmental, climatic, and healthcare-access factors in shaping urticaria disparities.

Our analysis reveals a complex relationship between urticaria burden and SDI in children and adolescents. We found a negative correlation between SDI and urticaria burden, with higher-SDI regions showing lower prevalence, incidence, and DALY rates. This pattern may stem from higher infectious disease burdens and environmental pollution in lower-SDI regions,^[34]^ which are undergoing industrialization and urbanization. These processes introduce new environmental allergens, medications, and food additives, all potential urticaria triggers.^[22,35]^ The economic implications are substantial, with studies showing that annual healthcare costs for children with urticaria average $2090 higher than for healthy children,^[36]^ creating significant challenges for lower-GDP regions. The heterogeneous distribution of urticaria burden across SDI strata underscores the critical need for tailored public health interventions that consider regional developmental contexts and specific risk factors.^[37]^

Our analysis reveals significant age-specific variations in pediatric urticaria burden. Children under 5 years have the highest incidence, prevalence, and DALY rates, which decline progressively with age. This pattern likely reflects immune system maturation and its relationship with infectious triggers. Infections, the most common cause of acute urticaria in children, account for up to 48.4% of cases.^[38]^ The immature immune systems of infants and young children are highly susceptible to pathogens and allergens, which can trigger acute urticaria. Emerging evidence suggests that recurrent infections may not only exacerbate existing symptoms but also contribute to the progression from acute to chronic CSU in some children.^[39]^ As children mature, their immune systems develop greater tolerance to environmental allergens and infections, correlating with the decline in urticaria burden during adolescence. These findings highlight the need for age-tailored prevention strategies, focusing on early childhood infection control and allergen management across developmental stages to reduce disease occurrence and burden.

Our findings align with prior studies,^[26,40]^ revealing that females have significantly higher incidence, prevalence, and DALY rates of urticaria than males. This sex difference may be partly explained by hormonal and immunological mechanisms: estrogen can enhance mast cell activation and histamine release,^[41]^ while X-linked immune genes may contribute to greater immune responsiveness in females,^[42]^These factors together may increase urticaria susceptibility, particularly during adolescence.

Our decomposition analysis shows that the urticaria burden among individuals under 20 years increased from 1990 to 2021 across different SDI regions. Epidemiological changes positively impacted urticaria prevalence in all SDI regions, likely due to increased exposure to allergens and irritants (e.g., air pollution, chemicals),^[35]^ changes in lifestyle factors (e.g., diet, stress, sedentary behavior),^[43,44]^ and improved diagnostic practices.^[45]^ These factors may have contributed to higher detection rates of urticaria. Conversely, population changes, such as declining birth rates and aging populations,^[46]^ while children and adolescents are the primary population affected by urticaria.

The BAPC modeling forecasts a persistent upward trend in urticaria’s global incidence, prevalence, and DALY rates until 2050, with female pediatric populations expected to experience disproportionately greater disease burden. These projections are model-based and reflect assumptions of continued historical trends rather than direct observations. This highlights the critical necessity for implementing sex-specific preventive interventions focused on addressable risk factors during key developmental periods.

This study has several limitations. First, the analysis relied on modeled GBD 2021 estimates, which lack primary validation across all contexts. Potential underdiagnosis and data gaps in low- and middle-income countries may lead to underestimation of disease burden. Furthermore, the dataset did not distinguish between acute and chronic urticaria or specific subtypes (such as dermatographic or cold urticaria), preventing subtype-specific epidemiological analysis. Second, the study was descriptive in nature and did not investigate underlying risk factors or causes. Finally, most cases were diagnosed by emergency physicians rather than dermatologists, which may introduce diagnostic bias due to differing levels of expertise.

## 5. Conclusions

This study, using GBD 2021 data, shows an increasing burden of urticaria among children and adolescents from 1990 to 2021, with projected rises in incidence, prevalence, and DALY rates by 2050. Females consistently have higher urticaria burdens than males, and significant health disparities related to SDI are evident. Lower SDI regions and children under 5, who have the highest incidence and prevalence rates, require targeted interventions. Early diagnosis, optimized treatment, and global health policy development are crucial to address these disparities.

## Acknowledgments

This study hereby acknowledges the global burden of disease (GBD) database for its open data resources and support.

## Author contributions

**Methodology:** Songhui Liu, Xiaohui Jing.

**Software:** Songhui Liu, Ting He.

**Validation:** Songhui Liu, Xiaohui Jing, Ting He.

**Visualization:** Songhui Liu, Fucheng Feng.

**Writing – original draft:** Songhui Liu, Xiaohui Jing.

**Writing – review & editing:** Songhui Liu, Ting He.

**Funding acquisition:** Xiaohui Jing, Ting He.

**Investigation:** Xiaohui Jing, Ting He.

**Resources:** Fucheng Feng, Ting He.

**Supervision:** Fucheng Feng, Ting He.

**Project administration:** Honglin Wang.

## Supplementary Material


