# Supplementary material for: Global burden of urticaria in children and adolescents, 1990 to 2021: Trends, disparities, and future projections
Source: Medicine (Baltimore). 2026 Jan 30;105(5):e47488. doi: 10.1097/MD.0000000000047488 (PMC12863908; doi:10.1097/MD.0000000000047488)
Supplement: Supplementary file 1 [file medi-105-e47488-s001.docx]

**Supplementary Material**

**Supplementary Table 1. Age-standardized prevalence, incidence, and DALYs of urticaria in children and adolescents aged <20 years (1990–2021) and EAPCs in 204 countries and territories.**

|  | **Prevalence (95% UI)** | | **Incidence (95% UI)** | | **DALYs (95% UI)** | |
| --- | --- | --- | --- | --- | --- | --- |
|  | **ASR in 2021 (per 100, 000population)** | **EAPC (1990–2021)** | **ASR in 2021 (per 100, 000population)** | **EAPC (1990–2021)** | **ASR in 2021 (per 100, 000population)** | EAPC (1990–2021） |
|  |  |  |  |  |  |  |
| Afghanistan | 1224.17 (1082.24, 1366.10) | 0.0018 (-0.0023, 0.0058) | 2185.64 (1893.92, 2477.36) | -0.0221 (-0.0271, -0.0170) | 75.12 (66.26, 83.98) | 0.0251 (0.0185, 0.0317) |
| Albania | 1749.23 (1485.95, 2012.51) | -0.0164 (-0.0234, -0.0094) | 3083.67 (2564.31, 3603.03) | -0.0037 (-0.0074, 0.0001) | 108.09 (91.68, 124.50) | -0.0002 (-0.0081, 0.0077) |
| Algeria | 1230.64 (1086.94, 1374.34) | -0.0105 (-0.0163, -0.0048) | 2183.50 (1894.46, 2472.54) | 0.0022 (-0.0046, 0.0090) | 75.68 (66.67, 84.69) | 0.0077 (0.0001, 0.0154) |
| American Samoa | 964.93 (905.93, 1023.93) | 0.0291 (0.0272, 0.0310) | 1723.94 (1597.07, 1850.81) | -0.0014 (-0.0027, -0.0001) | 59.32 (55.56, 63.08) | 0.0215 (0.0151, 0.0279) |
| Andorra | 467.99 (426.25, 509.73) | 0.0083 (0.0033, 0.0134) | 838.55 (756.79, 920.31) | -0.0252 (-0.0372, -0.0132) | 28.90 (26.30, 31.50) | 0.0146 (0.0052, 0.0240) |
| Angola | 1143.23 (1012.76, 1273.70) | 0.0216 (0.0207, 0.0224) | 2036.69 (1769.52, 2303.86) | -0.0102 (-0.0135, -0.0069) | 70.32 (62.25, 78.39) | 0.0506 (0.0443, 0.0568) |
| Antigua and Barbuda | 1141.01 (1009.73, 1272.29) | -0.0114 (-0.0146, -0.0082) | 2027.59 (1761.26, 2293.92) | -0.0138 (-0.0171, -0.0104) | 69.94 (61.77, 78.11) | -0.0076 (-0.0136, -0.0016) |
| Argentina | 1033.16 (913.25, 1153.07) | -0.0003 (-0.0052, 0.0046) | 1823.71 (1587.08, 2060.34) | -0.0060 (-0.0116, -0.0004) | 63.55 (56.11, 70.99) | -0.0020 (-0.0100, 0.0060) |
| Armenia | 1680.22 (1438.93, 1921.51) | -0.0194 (-0.0261, -0.0127) | 2957.17 (2481.85, 3432.49) | -0.0033 (-0.0121, 0.0055) | 103.28 (88.36, 118.20) | -0.0047 (-0.0120, 0.0027) |
| Australia | 1109.07 (999.68, 1218.46) | -0.0034 (-0.0064, -0.0004) | 1974.62 (1750.87, 2198.37) | 0.0000 (-0.0031, 0.0031) | 68.21 (61.31, 75.11) | 0.0034 (-0.0007, 0.0076) |
| Austria | 466.90 (425.47, 508.33) | -0.0057 (-0.0083, -0.0031) | 840.06 (757.16, 922.96) | -0.0010 (-0.0068, 0.0048) | 28.63 (26.05, 31.21) | 0.0001 (-0.0089, 0.0090) |
| Azerbaijan | 1679.77 (1438.42, 1921.12) | -0.0178 (-0.0256, -0.0101) | 2954.21 (2479.70, 3428.72) | -0.0046 (-0.0134, 0.0042) | 103.35 (88.48, 118.22) | -0.0061 (-0.0159, 0.0037) |
| Bahamas | 1144.67 (1013.03, 1276.31) | 0.0104 (0.0021, 0.0188) | 2028.53 (1763.84, 2293.22) | -0.0018 (-0.0091, 0.0055) | 70.27 (62.06, 78.48) | 0.0121 (0.0018, 0.0224) |
| Bahrain | 1226.68 (1080.56, 1372.80) | -0.0212 (-0.0310, -0.0115) | 2174.40 (1882.63, 2466.17) | -0.0278 (-0.0379, -0.0178) | 75.63 (66.55, 84.71) | -0.0061 (-0.0168, 0.0046) |
| Bangladesh | 1606.56 (1428.02, 1785.10) | 0.0255 (0.0194, 0.0316) | 2849.34 (2487.93, 3210.75) | 0.0078 (0.0033, 0.0122) | 98.84 (87.79, 109.89) | 0.0656 (0.0583, 0.0730) |
| Barbados | 1140.79 (1009.28, 1272.30) | 0.0003 (-0.0035, 0.0040) | 2023.89 (1758.56, 2289.22) | -0.0132 (-0.0168, -0.0097) | 70.07 (61.91, 78.23) | 0.0012 (-0.0055, 0.0079) |
| Belarus | 1685.92 (1444.47, 1927.37) | -0.0125 (-0.0222, -0.0029) | 2960.43 (2487.92, 3432.94) | 0.0093 (-0.0009, 0.0195) | 104.37 (89.31, 119.43) | -0.0088 (-0.0202, 0.0027) |
| Belgium | 467.95 (426.50, 509.40) | 0.0023 (0.0010, 0.0037) | 841.24 (758.67, 923.81) | -0.0046 (-0.0098, 0.0006) | 28.85 (26.25, 31.45) | 0.0064 (-0.0036, 0.0164) |
| Belize | 1142.86 (1011.69, 1274.03) | 0.0069 (0.0049, 0.0090) | 2030.93 (1764.70, 2297.16) | -0.0010 (-0.0026, 0.0006) | 70.14 (62.02, 78.26) | 0.0159 (0.0108, 0.0209) |
| Benin | 1139.57 (1009.94, 1269.20) | 0.0144 (0.0138, 0.0150) | 2034.51 (1767.03, 2301.99) | 0.0056 (0.0041, 0.0072) | 69.56 (61.54, 77.58) | 0.0329 (0.0260, 0.0398) |
| Bermuda | 1142.23 (1010.71, 1273.75) | 0.0033 (0.0013, 0.0054) | 2026.70 (1761.24, 2292.16) | -0.0085 (-0.0118, -0.0053) | 70.39 (62.20, 78.58) | 0.0104 (0.0036, 0.0172) |
| Bhutan | 1606.00 (1426.84, 1785.16) | 0.0256 (0.0211, 0.0302) | 2854.84 (2489.69, 3219.99) | 0.0100 (0.0070, 0.0130) | 98.23 (87.27, 109.19) | 0.0481 (0.0422, 0.0539) |
| Bolivia (Plurinational State of) | 1140.51 (1009.64, 1271.38) | 0.0021 (0.0002, 0.0040) | 2028.56 (1762.15, 2294.97) | -0.0045 (-0.0064, -0.0026) | 69.85 (61.81, 77.89) | 0.0292 (0.0227, 0.0357) |
| Bosnia and Herzegovina | 1755.38 (1491.44, 2019.32) | -0.0077 (-0.0125, -0.0029) | 3083.87 (2568.15, 3599.59) | -0.0015 (-0.0062, 0.0032) | 108.18 (91.76, 124.60) | -0.0020 (-0.0099, 0.0059) |
| Botswana | 1141.31 (1010.33, 1272.29) | -0.0073 (-0.0082, -0.0065) | 2030.30 (1763.56, 2297.04) | -0.0142 (-0.0147, -0.0136) | 70.03 (61.88, 78.18) | 0.0033 (-0.0016, 0.0083) |
| Brazil | 1200.70 (1068.39, 1333.01) | -0.0085 (-0.0109, -0.0060) | 2136.12 (1865.97, 2406.27) | -0.0018 (-0.0031, -0.0004) | 73.55 (65.32, 81.78) | 0.0069 (0.0040, 0.0098) |
| Brunei Darussalam | 1026.34 (906.40, 1146.28) | -0.0125 (-0.0202, -0.0047) | 1822.83 (1581.03, 2064.63) | -0.0154 (-0.0189, -0.0119) | 63.38 (55.89, 70.87) | -0.0083 (-0.0195, 0.0029) |
| Bulgaria | 1753.23 (1489.97, 2016.49) | -0.0087 (-0.0152, -0.0023) | 3089.21 (2570.64, 3607.78) | 0.0089 (-0.0000, 0.0179) | 108.28 (91.91, 124.65) | -0.0034 (-0.0103, 0.0035) |
| Burkina Faso | 1140.12 (1010.92, 1269.32) | 0.0129 (0.0112, 0.0145) | 2039.04 (1770.79, 2307.29) | 0.0042 (0.0036, 0.0047) | 69.18 (61.34, 77.02) | 0.0465 (0.0365, 0.0565) |
| Burundi | 1143.54 (1013.18, 1273.90) | 0.0077 (0.0056, 0.0098) | 2038.38 (1770.90, 2305.86) | -0.0048 (-0.0091, -0.0006) | 69.90 (61.89, 77.91) | 0.0468 (0.0375, 0.0560) |
| Cabo Verde | 1136.44 (1006.02, 1266.86) | -0.0035 (-0.0053, -0.0017) | 2026.62 (1758.86, 2294.38) | -0.0146 (-0.0165, -0.0126) | 69.37 (61.34, 77.40) | 0.0231 (0.0155, 0.0307) |
| Cambodia | 1142.96 (1011.39, 1274.53) | 0.0024 (0.0007, 0.0040) | 2027.54 (1762.15, 2292.93) | -0.0075 (-0.0091, -0.0059) | 70.22 (62.05, 78.39) | 0.0169 (0.0115, 0.0224) |
| Cameroon | 964.36 (941.64, 987.08) | 0.0030 (0.0006, 0.0055) | 1742.24 (1692.66, 1791.82) | -0.0001 (-0.0002, -0.0000) | 59.23 (57.83, 60.63) | 0.0388 (0.0318, 0.0458) |
| Canada | 1139.61 (1009.28, 1269.94) | 0.0071 (0.0051, 0.0091) | 2030.09 (1763.31, 2296.87) | -0.0082 (-0.0107, -0.0058) | 69.82 (61.73, 77.91) | 0.0353 (0.0269, 0.0437) |
| Central African Republic | 1312.07 (1148.73, 1475.41) | -0.0005 (-0.0041, 0.0030) | 2313.56 (1990.01, 2637.11) | -0.0015 (-0.0062, 0.0031) | 81.04 (70.76, 91.32) | 0.0019 (-0.0036, 0.0075) |
| Chad | 1139.24 (1009.56, 1268.92) | 0.0082 (0.0073, 0.0092) | 2034.10 (1766.51, 2301.69) | -0.0064 (-0.0080, -0.0048) | 69.20 (61.26, 77.14) | 0.0380 (0.0292, 0.0469) |
| Chile | 1137.75 (1008.83, 1266.67) | 0.0029 (0.0018, 0.0041) | 2036.72 (1768.24, 2305.20) | -0.0106 (-0.0116, -0.0096) | 69.47 (61.55, 77.39) | 0.0177 (0.0120, 0.0234) |
| China | 1031.07 (911.32, 1150.82) | -0.0005 (-0.0046, 0.0037) | 1824.98 (1586.39, 2063.57) | -0.0069 (-0.0126, -0.0011) | 63.56 (56.04, 71.08) | -0.0002 (-0.0072, 0.0067) |
| Colombia | 980.95 (932.16, 1029.74) | -0.0160 (-0.0267, -0.0053) | 1748.22 (1644.66, 1851.78) | -0.0244 (-0.0277, -0.0210) | 60.68 (57.58, 63.78) | -0.0034 (-0.0132, 0.0064) |
| Comoros | 1140.33 (1008.96, 1271.70) | -0.0021 (-0.0039, -0.0002) | 2025.02 (1759.14, 2290.90) | -0.0121 (-0.0147, -0.0094) | 70.34 (62.10, 78.58) | 0.0123 (0.0057, 0.0190) |
| Congo | 1140.00 (1009.41, 1270.59) | 0.0079 (0.0066, 0.0091) | 2029.06 (1762.56, 2295.56) | -0.0120 (-0.0126, -0.0114) | 69.86 (61.76, 77.96) | 0.0317 (0.0254, 0.0380) |
| Cook Islands | 1143.14 (1012.02, 1274.26) | 0.0085 (0.0059, 0.0112) | 2031.10 (1765.09, 2297.11) | -0.0174 (-0.0216, -0.0133) | 69.88 (61.77, 77.99) | 0.0269 (0.0175, 0.0363) |
| Costa Rica | 964.22 (906.97, 1021.47) | 0.0162 (0.0122, 0.0201) | 1727.90 (1602.00, 1853.80) | 0.0105 (0.0071, 0.0138) | 59.57 (55.99, 63.15) | 0.0249 (0.0191, 0.0308) |
| Croatia | 1144.85 (1012.42, 1277.28) | 0.0071 (0.0041, 0.0101) | 2022.89 (1759.33, 2286.45) | 0.0025 (-0.0010, 0.0061) | 70.47 (62.23, 78.71) | 0.0049 (-0.0017, 0.0115) |
| Cuba | 1753.45 (1490.28, 2016.62) | -0.0053 (-0.0097, -0.0009) | 3090.38 (2571.63, 3609.13) | 0.0033 (-0.0031, 0.0097) | 108.31 (91.91, 124.71) | 0.0051 (-0.0006, 0.0108) |
| Cyprus | 1140.58 (1008.76, 1272.40) | -0.0032 (-0.0088, 0.0023) | 2019.37 (1755.29, 2283.45) | -0.0015 (-0.0064, 0.0033) | 70.21 (62.02, 78.40) | 0.0030 (-0.0071, 0.0131) |
| Czechia | 466.76 (425.32, 508.20) | 0.0037 (0.0017, 0.0058) | 841.06 (757.55, 924.57) | 0.0103 (0.0018, 0.0189) | 28.78 (26.14, 31.42) | 0.0162 (0.0073, 0.0251) |
| Côte d'Ivoire | 1754.74 (1491.22, 2018.26) | -0.0075 (-0.0147, -0.0003) | 3089.79 (2571.70, 3607.88) | 0.0096 (-0.0000, 0.0192) | 108.51 (92.06, 124.96) | -0.0006 (-0.0082, 0.0070) |
| Democratic People's Republic of Korea | 958.52 (907.43, 1009.61) | -0.0053 (-0.0094, -0.0012) | 1722.08 (1607.06, 1837.10) | -0.0216 (-0.0276, -0.0156) | 59.13 (55.93, 62.33) | 0.0011 (-0.0049, 0.0071) |
| Democratic Republic of the Congo | 1140.13 (1009.39, 1270.87) | 0.0108 (0.0087, 0.0130) | 2029.67 (1762.76, 2296.58) | -0.0133 (-0.0145, -0.0120) | 69.60 (61.52, 77.68) | 0.0485 (0.0400, 0.0569) |
| Denmark | 586.26 (525.10, 647.42) | 0.0013 (-0.0011, 0.0036) | 1035.84 (917.22, 1154.46) | -0.0126 (-0.0197, -0.0056) | 36.05 (32.23, 39.87) | 0.0067 (-0.0002, 0.0137) |
| Djibouti | 1128.76 (997.97, 1259.55) | -0.0101 (-0.0169, -0.0032) | 2009.57 (1742.85, 2276.29) | -0.0254 (-0.0281, -0.0227) | 69.34 (61.29, 77.39) | 0.0126 (0.0036, 0.0216) |
| Dominica | 1143.25 (1010.87, 1275.63) | 0.0169 (0.0092, 0.0247) | 2023.48 (1758.63, 2288.33) | -0.0082 (-0.0105, -0.0058) | 70.16 (61.93, 78.39) | 0.0147 (0.0063, 0.0231) |
| Dominican Republic | 1140.10 (1009.42, 1270.78) | -0.0049 (-0.0067, -0.0031) | 2029.03 (1762.45, 2295.61) | -0.0127 (-0.0163, -0.0091) | 70.01 (61.86, 78.16) | 0.0045 (-0.0002, 0.0092) |
| Ecuador | 1142.11 (1010.54, 1273.68) | 0.0051 (0.0024, 0.0078) | 2026.16 (1760.71, 2291.61) | -0.0091 (-0.0116, -0.0066) | 70.42 (62.18, 78.66) | 0.0252 (0.0197, 0.0306) |
| Egypt | 1467.96 (1301.73, 1634.19) | -0.0029 (-0.0084, 0.0026) | 2578.98 (2246.42, 2911.54) | -0.0027 (-0.0104, 0.0051) | 90.46 (80.08, 100.84) | 0.0208 (0.0137, 0.0278) |
| El Salvador | 1139.86 (1008.81, 1270.91) | -0.0016 (-0.0074, 0.0041) | 2022.91 (1758.12, 2287.70) | -0.0163 (-0.0203, -0.0123) | 70.28 (62.12, 78.44) | 0.0251 (0.0154, 0.0348) |
| Equatorial Guinea | 1125.25 (993.49, 1257.01) | -0.0337 (-0.0405, -0.0269) | 1999.37 (1732.76, 2265.98) | -0.0669 (-0.0723, -0.0614) | 69.01 (60.88, 77.14) | 0.0255 (0.0202, 0.0307) |
| Eritrea | 1136.57 (1006.20, 1266.94) | 0.0093 (0.0077, 0.0110) | 2025.08 (1758.19, 2291.97) | -0.0026 (-0.0039, -0.0013) | 69.57 (61.52, 77.62) | 0.0380 (0.0321, 0.0439) |
| Estonia | 1684.36 (1443.91, 1924.81) | -0.0159 (-0.0265, -0.0053) | 2967.04 (2492.26, 3441.82) | 0.0160 (0.0057, 0.0262) | 103.78 (88.83, 118.73) | -0.0072 (-0.0168, 0.0023) |
| Eswatini | 1138.13 (1007.33, 1268.93) | -0.0146 (-0.0178, -0.0115) | 2025.17 (1758.56, 2291.78) | -0.0233 (-0.0290, -0.0175) | 69.70 (61.56, 77.84) | -0.0069 (-0.0149, 0.0010) |
| Ethiopia | 1197.85 (1066.45, 1329.25) | 0.0150 (0.0128, 0.0171) | 2137.84 (1866.58, 2409.10) | -0.0082 (-0.0095, -0.0070) | 73.22 (65.12, 81.32) | 0.0464 (0.0429, 0.0499) |
| Fiji | 963.01 (905.24, 1020.78) | -0.0007 (-0.0049, 0.0035) | 1726.63 (1599.64, 1853.62) | 0.0016 (0.0009, 0.0022) | 59.17 (55.57, 62.77) | 0.0069 (-0.0001, 0.0139) |
| Finland | 467.62 (426.31, 508.93) | 0.0013 (-0.0007, 0.0033) | 842.02 (759.05, 924.99) | -0.0098 (-0.0167, -0.0030) | 28.80 (26.21, 31.39) | 0.0021 (-0.0069, 0.0110) |
| France | 619.51 (568.83, 670.19) | 0.0001 (-0.0011, 0.0014) | 1096.15 (995.79, 1196.51) | -0.0029 (-0.0066, 0.0008) | 37.86 (34.64, 41.08) | -0.0046 (-0.0107, 0.0015) |
| Gabon | 1147.43 (1016.72, 1278.14) | 0.0205 (0.0190, 0.0220) | 2038.60 (1773.22, 2303.98) | 0.0018 (-0.0019, 0.0055) | 70.08 (61.96, 78.20) | 0.0512 (0.0447, 0.0576) |
| Gambia | 1140.91 (1010.95, 1270.87) | 0.0130 (0.0115, 0.0146) | 2034.33 (1767.34, 2301.32) | -0.0119 (-0.0142, -0.0095) | 69.69 (61.70, 77.68) | 0.0292 (0.0235, 0.0348) |
| Georgia | 1681.29 (1439.73, 1922.85) | -0.0263 (-0.0332, -0.0195) | 2957.12 (2482.24, 3432.00) | 0.0046 (-0.0030, 0.0122) | 103.65 (88.77, 118.53) | -0.0198 (-0.0285, -0.0111) |
| Germany | 384.21 (348.01, 420.41) | -0.0067 (-0.0089, -0.0046) | 697.67 (624.94, 770.40) | 0.0040 (-0.0019, 0.0099) | 23.66 (21.36, 25.96) | 0.0022 (-0.0076, 0.0120) |
| Ghana | 1139.79 (1009.19, 1270.39) | 0.0048 (0.0036, 0.0061) | 2028.72 (1762.19, 2295.25) | -0.0032 (-0.0053, -0.0011) | 69.68 (61.68, 77.68) | 0.0342 (0.0282, 0.0401) |
| Greece | 557.66 (507.86, 607.46) | 0.0065 (0.0016, 0.0114) | 989.58 (891.37, 1087.79) | 0.0130 (0.0077, 0.0183) | 34.24 (31.09, 37.39) | 0.0048 (-0.0031, 0.0127) |
| Greenland | 1314.53 (1151.28, 1477.78) | 0.0094 (0.0035, 0.0152) | 2317.39 (1994.20, 2640.58) | 0.0118 (0.0048, 0.0188) | 81.13 (70.88, 91.38) | 0.0164 (0.0103, 0.0225) |
| Grenada | 1138.92 (1007.34, 1270.50) | -0.0178 (-0.0229, -0.0127) | 2023.05 (1756.65, 2289.45) | -0.0219 (-0.0297, -0.0141) | 69.84 (61.67, 78.01) | -0.0128 (-0.0204, -0.0052) |
| Guam | 960.97 (903.66, 1018.28) | 0.0008 (-0.0048, 0.0064) | 1726.03 (1598.78, 1853.28) | -0.0066 (-0.0087, -0.0045) | 59.31 (55.68, 62.94) | -0.0055 (-0.0148, 0.0037) |
| Guatemala | 1144.09 (1012.28, 1275.90) | 0.0072 (0.0054, 0.0089) | 2027.61 (1762.58, 2292.64) | -0.0140 (-0.0160, -0.0121) | 70.27 (62.04, 78.50) | 0.0329 (0.0248, 0.0409) |
| Guinea | 1139.72 (1010.00, 1269.44) | 0.0106 (0.0077, 0.0134) | 2033.07 (1766.14, 2300.00) | -0.0059 (-0.0096, -0.0022) | 69.45 (61.49, 77.41) | 0.0215 (0.0169, 0.0261) |
| Guinea-Bissau | 1140.04 (1009.84, 1270.24) | 0.0024 (0.0008, 0.0039) | 2032.08 (1765.00, 2299.16) | -0.0135 (-0.0160, -0.0110) | 69.83 (61.81, 77.85) | 0.0300 (0.0220, 0.0381) |
| Guyana | 1141.04 (1010.10, 1271.98) | -0.0033 (-0.0065, -0.0000) | 2028.01 (1762.14, 2293.88) | -0.0097 (-0.0159, -0.0036) | 70.05 (61.88, 78.22) | 0.0151 (0.0100, 0.0202) |
| Haiti | 1140.39 (1010.08, 1270.70) | -0.0062 (-0.0079, -0.0046) | 2033.34 (1765.79, 2300.89) | -0.0154 (-0.0159, -0.0149) | 69.13 (61.13, 77.13) | -0.0081 (-0.0132, -0.0030) |
| Honduras | 1140.66 (1009.82, 1271.50) | 0.0036 (0.0022, 0.0050) | 2028.34 (1762.17, 2294.51) | -0.0039 (-0.0046, -0.0033) | 70.18 (62.01, 78.35) | 0.0238 (0.0178, 0.0297) |
| Hungary | 1753.05 (1490.07, 2016.03) | -0.0059 (-0.0092, -0.0026) | 3092.03 (2572.58, 3611.48) | 0.0036 (-0.0009, 0.0082) | 108.14 (91.80, 124.48) | 0.0014 (-0.0042, 0.0071) |
| Iceland | 467.65 (426.45, 508.85) | 0.0011 (-0.0016, 0.0037) | 845.08 (760.87, 929.29) | -0.0008 (-0.0088, 0.0072) | 28.83 (26.24, 31.42) | 0.0007 (-0.0070, 0.0084) |
| India | 1329.30 (1166.67, 1491.93) | 0.0070 (0.0043, 0.0097) | 2361.73 (2034.09, 2689.37) | -0.0091 (-0.0108, -0.0074) | 81.08 (71.12, 91.04) | 0.0321 (0.0297, 0.0346) |
| Indonesia | 1005.24 (979.70, 1030.78) | 0.0030 (0.0022, 0.0037) | 1815.38 (1760.67, 1870.09) | 0.0005 (0.0003, 0.0006) | 61.85 (60.23, 63.47) | 0.0236 (0.0212, 0.0261) |
| Iran (Islamic Republic of) | 1304.18 (1157.72, 1450.64) | -0.0137 (-0.0218, -0.0057) | 2303.40 (2013.27, 2593.53) | 0.0004 (-0.0070, 0.0077) | 80.22 (71.06, 89.38) | -0.0008 (-0.0083, 0.0067) |
| Iraq | 1229.75 (1085.87, 1373.63) | 0.0101 (0.0082, 0.0120) | 2181.78 (1892.53, 2471.03) | -0.0100 (-0.0124, -0.0077) | 75.64 (66.64, 84.64) | 0.0208 (0.0146, 0.0271) |
| Ireland | 468.13 (426.65, 509.61) | 0.0042 (0.0013, 0.0071) | 840.76 (758.53, 922.99) | 0.0013 (-0.0077, 0.0103) | 28.77 (26.15, 31.39) | 0.0151 (0.0069, 0.0233) |
| Israel | 422.70 (385.66, 459.74) | 0.0029 (0.0021, 0.0037) | 765.61 (690.31, 840.91) | 0.0019 (-0.0011, 0.0049) | 26.12 (23.76, 28.48) | 0.0041 (-0.0046, 0.0128) |
| Italy | 323.27 (298.82, 347.72) | -0.0016 (-0.0029, -0.0002) | 587.87 (538.25, 637.49) | -0.0191 (-0.0235, -0.0148) | 19.83 (18.30, 21.36) | -0.0056 (-0.0085, -0.0027) |
| Jamaica | 1142.37 (1010.62, 1274.12) | -0.0029 (-0.0051, -0.0007) | 2026.39 (1760.74, 2292.04) | -0.0093 (-0.0118, -0.0068) | 70.13 (61.97, 78.29) | 0.0019 (-0.0043, 0.0082) |
| Japan | 1086.81 (966.46, 1207.16) | 0.0015 (-0.0006, 0.0036) | 1929.08 (1686.56, 2171.60) | -0.0030 (-0.0052, -0.0008) | 67.03 (59.51, 74.55) | 0.0042 (0.0023, 0.0061) |
| Jordan | 1229.40 (1085.37, 1373.43) | 0.0066 (0.0041, 0.0091) | 2181.94 (1892.19, 2471.69) | -0.0168 (-0.0201, -0.0134) | 75.67 (66.69, 84.65) | 0.0193 (0.0117, 0.0269) |
| Kazakhstan | 1679.64 (1441.47, 1917.81) | -0.0191 (-0.0279, -0.0102) | 2978.39 (2499.16, 3457.62) | 0.0179 (0.0082, 0.0277) | 103.44 (88.64, 118.24) | 0.0014 (-0.0069, 0.0098) |
| Kenya | 1202.64 (1069.85, 1335.43) | 0.0098 (0.0078, 0.0117) | 2137.34 (1867.25, 2407.43) | -0.0143 (-0.0150, -0.0135) | 73.87 (65.63, 82.11) | 0.0304 (0.0262, 0.0346) |
| Kiribati | 962.66 (905.42, 1019.90) | 0.0064 (0.0037, 0.0090) | 1727.32 (1600.73, 1853.91) | -0.0014 (-0.0022, -0.0005) | 59.07 (55.56, 62.58) | 0.0282 (0.0216, 0.0347) |
| Kuwait | 1230.94 (1086.69, 1375.19) | -0.0049 (-0.0171, 0.0072) | 2181.23 (1892.45, 2470.01) | -0.0109 (-0.0186, -0.0032) | 75.78 (66.75, 84.81) | -0.0054 (-0.0183, 0.0075) |
| Kyrgyzstan | 1683.13 (1443.57, 1922.69) | -0.0124 (-0.0200, -0.0048) | 2971.21 (2495.29, 3447.13) | 0.0092 (0.0009, 0.0176) | 99.03 (87.63, 110.43) | -0.0422 (-0.1267, 0.0423) |
| Lao People's Democratic Republic | 963.62 (940.81, 986.43) | 0.0067 (0.0047, 0.0087) | 1742.30 (1692.67, 1791.93) | -0.0001 (-0.0003, 0.0000) | 59.14 (57.70, 60.58) | 0.0289 (0.0216, 0.0363) |
| Latvia | 1685.59 (1444.46, 1926.72) | -0.0154 (-0.0245, -0.0062) | 2962.04 (2489.14, 3434.94) | 0.0146 (0.0037, 0.0255) | 104.16 (89.08, 119.24) | -0.0088 (-0.0190, 0.0015) |
| Lebanon | 1224.00 (1080.25, 1367.75) | -0.0004 (-0.0060, 0.0052) | 2173.95 (1884.07, 2463.83) | -0.0198 (-0.0236, -0.0159) | 75.29 (66.31, 84.27) | 0.0124 (0.0070, 0.0177) |
| Lesotho | 1142.32 (1011.09, 1273.55) | 0.0033 (0.0017, 0.0049) | 2031.17 (1764.37, 2297.97) | -0.0058 (-0.0072, -0.0044) | 69.98 (61.79, 78.17) | 0.0120 (0.0057, 0.0183) |
| Liberia | 1139.58 (1009.25, 1269.91) | 0.0053 (-0.0002, 0.0107) | 2029.69 (1763.07, 2296.31) | -0.0231 (-0.0263, -0.0198) | 69.28 (61.28, 77.28) | 0.0358 (0.0260, 0.0456) |
| Libya | 1232.60 (1088.31, 1376.89) | -0.0267 (-0.0360, -0.0174) | 2183.72 (1895.02, 2472.42) | -0.0447 (-0.0523, -0.0370) | 75.75 (66.73, 84.77) | -0.0284 (-0.0387, -0.0182) |
| Lithuania | 1685.97 (1444.87, 1927.07) | -0.0116 (-0.0194, -0.0038) | 2963.52 (2490.34, 3436.70) | 0.0092 (0.0011, 0.0173) | 104.00 (88.99, 119.01) | -0.0088 (-0.0182, 0.0006) |
| Luxembourg | 466.88 (425.46, 508.30) | 0.0005 (-0.0021, 0.0030) | 841.67 (758.00, 925.34) | -0.0154 (-0.0190, -0.0119) | 28.84 (26.23, 31.45) | 0.0102 (-0.0007, 0.0211) |
| Madagascar | 1142.90 (1012.19, 1273.61) | 0.0164 (0.0158, 0.0170) | 2032.72 (1766.57, 2298.87) | -0.0074 (-0.0089, -0.0059) | 70.04 (61.97, 78.11) | 0.0419 (0.0195, 0.0643) |
| Malawi | 1143.81 (1012.98, 1274.64) | 0.0271 (0.0248, 0.0294) | 2035.67 (1768.75, 2302.59) | -0.0183 (-0.0211, -0.0154) | 69.90 (61.83, 77.97) | 0.0786 (0.0703, 0.0869) |
| Malaysia | 965.25 (942.62, 987.88) | 0.0043 (0.0019, 0.0067) | 1742.00 (1692.48, 1791.52) | 0.0002 (0.0001, 0.0003) | 59.20 (57.79, 60.61) | 0.0100 (0.0034, 0.0166) |
| Maldives | 965.89 (943.33, 988.45) | -0.0016 (-0.0068, 0.0035) | 1742.29 (1692.82, 1791.76) | -0.0001 (-0.0004, 0.0002) | 59.17 (57.77, 60.57) | 0.0335 (0.0254, 0.0415) |
| Mali | 1137.72 (1008.32, 1267.12) | 0.0080 (0.0071, 0.0089) | 2034.60 (1766.15, 2303.05) | -0.0086 (-0.0095, -0.0076) | 68.86 (61.01, 76.71) | 0.0217 (0.0161, 0.0273) |
| Malta | 467.21 (425.76, 508.66) | -0.0035 (-0.0052, -0.0017) | 841.12 (757.94, 924.30) | 0.0175 (0.0116, 0.0233) | 28.92 (26.32, 31.52) | -0.0020 (-0.0109, 0.0068) |
| Marshall Islands | 963.06 (905.49, 1020.63) | 0.0059 (0.0034, 0.0084) | 1727.48 (1600.56, 1854.40) | 0.0024 (0.0017, 0.0032) | 59.20 (55.63, 62.77) | 0.0120 (0.0062, 0.0177) |
| Mauritania | 1141.99 (1011.49, 1272.49) | 0.0153 (0.0141, 0.0166) | 2031.49 (1765.53, 2297.45) | -0.0019 (-0.0038, -0.0000) | 69.91 (61.79, 78.03) | 0.0414 (0.0368, 0.0460) |
| Mauritius | 965.08 (942.36, 987.80) | 0.0049 (0.0008, 0.0090) | 1742.52 (1692.84, 1792.20) | 0.0001 (-0.0001, 0.0003) | 59.17 (57.72, 60.62) | 0.0143 (0.0042, 0.0243) |
| Mexico | 1204.03 (1070.79, 1337.27) | 0.0052 (0.0027, 0.0077) | 2137.04 (1867.24, 2406.84) | -0.0086 (-0.0098, -0.0073) | 74.27 (65.95, 82.59) | 0.0139 (0.0106, 0.0172) |
| Micronesia (Federated States of) | 962.67 (904.73, 1020.61) | 0.0053 (0.0042, 0.0064) | 1725.43 (1598.36, 1852.50) | 0.0032 (0.0029, 0.0035) | 59.27 (55.65, 62.89) | 0.0154 (0.0063, 0.0245) |
| Monaco | 468.32 (426.88, 509.76) | 0.0239 (0.0204, 0.0275) | 842.81 (759.85, 925.77) | -0.0011 (-0.0088, 0.0067) | 28.78 (26.14, 31.42) | 0.0311 (0.0232, 0.0389) |
| Mongolia | 1683.50 (1443.73, 1923.27) | -0.0092 (-0.0177, -0.0008) | 2970.65 (2494.89, 3446.41) | 0.0067 (-0.0030, 0.0163) | 103.43 (88.64, 118.22) | 0.0093 (-0.0003, 0.0189) |
| Montenegro | 1750.70 (1487.57, 2013.83) | -0.0099 (-0.0117, -0.0080) | 3086.69 (2567.44, 3605.94) | -0.0006 (-0.0027, 0.0015) | 108.20 (91.80, 124.60) | -0.0094 (-0.0142, -0.0046) |
| Morocco | 1231.02 (1087.30, 1374.74) | -0.0077 (-0.0098, -0.0056) | 2186.34 (1896.30, 2476.38) | -0.0215 (-0.0251, -0.0178) | 75.63 (66.71, 84.55) | 0.0014 (-0.0041, 0.0070) |
| Mozambique | 1143.63 (1013.27, 1273.99) | 0.0199 (0.0177, 0.0222) | 2036.67 (1769.96, 2303.38) | -0.0106 (-0.0116, -0.0096) | 69.78 (61.72, 77.84) | 0.0581 (0.0504, 0.0659) |
| Myanmar | 964.37 (941.61, 987.13) | 0.0034 (0.0030, 0.0039) | 1742.26 (1692.62, 1791.90) | -0.0006 (-0.0006, -0.0006) | 59.03 (57.58, 60.48) | 0.0316 (0.0251, 0.0382) |
| Namibia | 1143.24 (1012.29, 1274.19) | 0.0039 (0.0019, 0.0059) | 2034.01 (1767.15, 2300.87) | -0.0046 (-0.0069, -0.0023) | 70.02 (61.91, 78.13) | 0.0174 (0.0104, 0.0244) |
| Nauru | 959.84 (902.68, 1017.00) | 0.0004 (-0.0023, 0.0030) | 1723.80 (1596.90, 1850.70) | -0.0077 (-0.0082, -0.0072) | 59.00 (55.41, 62.59) | 0.0048 (-0.0021, 0.0116) |
| Nepal | 2039.47 (1816.89, 2262.05) | 0.0087 (0.0036, 0.0139) | 3571.37 (3124.89, 4017.85) | -0.0114 (-0.0129, -0.0099) | 125.29 (111.48, 139.10) | 0.0410 (0.0335, 0.0485) |
| Netherlands | 467.43 (426.19, 508.67) | 0.0005 (-0.0011, 0.0020) | 843.50 (759.78, 927.22) | -0.0071 (-0.0112, -0.0029) | 28.82 (26.23, 31.41) | -0.0031 (-0.0095, 0.0033) |
| New Zealand | 1145.57 (1028.04, 1263.10) | 0.0018 (-0.0013, 0.0049) | 2041.98 (1801.74, 2282.22) | -0.0053 (-0.0089, -0.0018) | 70.41 (63.00, 77.82) | 0.0062 (0.0015, 0.0110) |
| Nicaragua | 1139.96 (1008.72, 1271.20) | -0.0082 (-0.0105, -0.0058) | 2024.28 (1758.65, 2289.91) | -0.0107 (-0.0125, -0.0089) | 70.12 (61.95, 78.29) | 0.0079 (0.0020, 0.0138) |
| Niger | 1138.11 (1009.05, 1267.17) | 0.0134 (0.0107, 0.0160) | 2035.89 (1767.74, 2304.04) | -0.0156 (-0.0177, -0.0134) | 69.64 (61.77, 77.51) | 0.0390 (0.0313, 0.0467) |
| Nigeria | 1203.84 (1072.95, 1334.73) | 0.0090 (-0.0012, 0.0193) | 2149.16 (1878.26, 2420.06) | -0.0176 (-0.0246, -0.0105) | 73.28 (65.26, 81.30) | 0.0381 (0.0235, 0.0527) |
| Niue | 960.68 (901.68, 1019.68) | -0.0012 (-0.0123, 0.0100) | 1721.19 (1592.81, 1849.57) | 0.0036 (-0.0069, 0.0141) | 59.25 (55.54, 62.96) | 0.0012 (-0.0092, 0.0116) |
| North Macedonia | 1753.38 (1489.81, 2016.95) | -0.0031 (-0.0064, 0.0001) | 3084.51 (2567.59, 3601.43) | -0.0015 (-0.0057, 0.0027) | 108.32 (91.93, 124.71) | 0.0005 (-0.0043, 0.0054) |
| Northern Mariana Islands | 962.83 (904.47, 1021.19) | 0.0090 (-0.0086, 0.0267) | 1722.39 (1595.75, 1849.03) | -0.0311 (-0.0397, -0.0225) | 59.24 (55.60, 62.88) | 0.0088 (-0.0075, 0.0251) |
| Norway | 500.88 (461.35, 540.41) | 0.0013 (-0.0003, 0.0029) | 904.31 (822.32, 986.30) | -0.0105 (-0.0143, -0.0068) | 30.83 (28.34, 33.32) | 0.0019 (-0.0018, 0.0056) |
| Oman | 1233.74 (1089.36, 1378.12) | 0.0018 (-0.0054, 0.0090) | 2186.26 (1897.15, 2475.37) | -0.0059 (-0.0092, -0.0025) | 75.86 (66.83, 84.89) | 0.0094 (-0.0002, 0.0190) |
| Pakistan | 1680.73 (1498.49, 1862.97) | 0.0039 (0.0019, 0.0059) | 2994.67 (2620.25, 3369.09) | -0.0033 (-0.0042, -0.0023) | 102.43 (91.29, 113.57) | 0.0234 (0.0211, 0.0258) |
| Palau | 962.82 (904.79, 1020.85) | -0.0029 (-0.0081, 0.0023) | 1723.67 (1597.24, 1850.10) | -0.0138 (-0.0169, -0.0107) | 59.39 (55.72, 63.06) | 0.0012 (-0.0069, 0.0094) |
| Palestine | 1230.89 (1087.16, 1374.62) | 0.0140 (0.0131, 0.0149) | 2185.54 (1895.74, 2475.34) | -0.0085 (-0.0111, -0.0060) | 75.76 (66.77, 84.75) | 0.0200 (0.0158, 0.0242) |
| Panama | 1140.98 (1009.35, 1272.61) | 0.0007 (-0.0022, 0.0035) | 2022.33 (1757.62, 2287.04) | -0.0071 (-0.0099, -0.0043) | 70.19 (62.01, 78.37) | 0.0100 (0.0035, 0.0165) |
| Papua New Guinea | 956.78 (899.96, 1013.60) | -0.0034 (-0.0053, -0.0015) | 1722.66 (1594.84, 1850.48) | -0.0014 (-0.0020, -0.0007) | 58.61 (55.08, 62.14) | 0.0159 (0.0104, 0.0215) |
| Paraguay | 1139.90 (1008.81, 1270.99) | -0.0028 (-0.0037, -0.0019) | 2024.87 (1759.22, 2290.52) | -0.0062 (-0.0069, -0.0055) | 69.94 (61.76, 78.12) | 0.0148 (0.0098, 0.0197) |
| Peru | 1136.02 (1005.03, 1267.01) | -0.0182 (-0.0215, -0.0150) | 2021.49 (1754.66, 2288.32) | -0.0202 (-0.0211, -0.0194) | 69.93 (61.80, 78.06) | 0.0076 (0.0016, 0.0136) |
| Philippines | 1004.75 (979.23, 1030.27) | 0.0040 (0.0024, 0.0056) | 1814.81 (1760.23, 1869.39) | -0.0001 (-0.0003, 0.0001) | 61.68 (60.06, 63.30) | 0.0204 (0.0179, 0.0229) |
| Poland | 2024.26 (1707.15, 2341.37) | -0.0255 (-0.0450, -0.0060) | 3555.35 (2940.23, 4170.47) | -0.0082 (-0.0290, 0.0127) | 124.81 (105.11, 144.51) | -0.0229 (-0.0412, -0.0045) |
| Portugal | 390.83 (354.51, 427.15) | -0.0001 (-0.0021, 0.0018) | 709.81 (636.71, 782.91) | -0.0067 (-0.0126, -0.0008) | 24.10 (21.81, 26.39) | 0.0011 (-0.0083, 0.0105) |
| Puerto Rico | 1143.73 (1011.21, 1276.25) | 0.0138 (0.0111, 0.0166) | 2019.90 (1756.67, 2283.13) | -0.0147 (-0.0163, -0.0131) | 70.26 (62.03, 78.49) | 0.0142 (0.0086, 0.0198) |
| Qatar | 1230.51 (1087.24, 1373.78) | -0.0036 (-0.0159, 0.0088) | 2194.29 (1901.19, 2487.39) | -0.0008 (-0.0118, 0.0101) | 75.87 (66.89, 84.85) | 0.0048 (-0.0078, 0.0174) |
| Republic of Korea | 1032.45 (911.88, 1153.02) | 0.0086 (0.0013, 0.0158) | 1819.95 (1583.22, 2056.68) | -0.0133 (-0.0211, -0.0055) | 63.73 (56.18, 71.28) | 0.0113 (0.0041, 0.0186) |
| Republic of Moldova | 1684.97 (1443.99, 1925.95) | -0.0104 (-0.0162, -0.0046) | 2963.52 (2489.79, 3437.25) | 0.0070 (0.0003, 0.0137) | 103.99 (88.99, 118.99) | -0.0024 (-0.0106, 0.0057) |
| Romania | 1616.69 (1374.95, 1858.43) | -0.0087 (-0.0162, -0.0012) | 2863.45 (2385.02, 3341.88) | 0.0153 (0.0077, 0.0229) | 99.64 (84.60, 114.68) | 0.0014 (-0.0049, 0.0078) |
| Russian Federation | 1762.74 (1520.41, 2005.07) | -0.0120 (-0.0232, -0.0007) | 3100.68 (2623.49, 3577.87) | 0.0081 (-0.0023, 0.0185) | 108.76 (93.70, 123.82) | -0.0022 (-0.0142, 0.0099) |
| Rwanda | 1141.56 (1011.15, 1271.97) | 0.0040 (-0.0014, 0.0094) | 2033.04 (1766.29, 2299.79) | -0.0183 (-0.0232, -0.0133) | 70.00 (61.92, 78.08) | 0.0533 (0.0426, 0.0639) |
| Saint Kitts and Nevis | 1144.02 (1012.24, 1275.80) | 0.0003 (-0.0033, 0.0039) | 2028.12 (1762.87, 2293.37) | -0.0001 (-0.0025, 0.0023) | 70.22 (62.02, 78.42) | 0.0053 (0.0005, 0.0101) |
| Saint Lucia | 1141.28 (1009.46, 1273.10) | -0.0128 (-0.0157, -0.0098) | 2023.59 (1758.23, 2288.95) | -0.0155 (-0.0174, -0.0136) | 70.04 (61.83, 78.25) | -0.0108 (-0.0165, -0.0051) |
| Saint Vincent and the Grenadines | 1143.91 (1011.31, 1276.51) | 0.0016 (-0.0022, 0.0053) | 2023.00 (1758.45, 2287.55) | -0.0093 (-0.0117, -0.0070) | 69.96 (61.72, 78.20) | 0.0096 (0.0030, 0.0162) |
| Samoa | 960.24 (903.09, 1017.39) | 0.0073 (0.0065, 0.0080) | 1725.67 (1598.34, 1853.00) | 0.0079 (0.0074, 0.0084) | 59.09 (55.57, 62.61) | 0.0078 (0.0022, 0.0135) |
| San Marino | 466.91 (425.38, 508.44) | -0.0103 (-0.0110, -0.0097) | 838.61 (756.22, 921.00) | -0.0384 (-0.0424, -0.0343) | 28.78 (26.18, 31.38) | -0.0050 (-0.0122, 0.0021) |
| Sao Tome and Principe | 1142.76 (1011.72, 1273.80) | 0.0195 (0.0160, 0.0230) | 2029.25 (1763.91, 2294.59) | -0.0085 (-0.0104, -0.0066) | 69.95 (61.84, 78.06) | 0.0394 (0.0323, 0.0466) |
| Saudi Arabia | 1221.82 (1076.38, 1367.26) | -0.0390 (-0.0501, -0.0280) | 2167.56 (1876.15, 2458.97) | -0.0419 (-0.0494, -0.0344) | 75.27 (66.15, 84.39) | -0.0257 (-0.0408, -0.0107) |
| Senegal | 1136.92 (1006.18, 1267.66) | -0.0147 (-0.0171, -0.0123) | 2025.57 (1758.07, 2293.07) | -0.0315 (-0.0339, -0.0291) | 69.24 (61.19, 77.29) | 0.0105 (0.0036, 0.0173) |
| Serbia | 1751.23 (1486.46, 2016.00) | -0.0169 (-0.0211, -0.0128) | 3074.38 (2557.98, 3590.78) | -0.0478 (-0.0550, -0.0406) | 108.12 (91.61, 124.63) | -0.0075 (-0.0146, -0.0004) |
| Seychelles | 964.63 (941.89, 987.37) | -0.0008 (-0.0034, 0.0018) | 1742.39 (1692.77, 1792.01) | 0.0005 (0.0003, 0.0006) | 59.39 (57.96, 60.82) | 0.0063 (0.0007, 0.0120) |
| Sierra Leone | 1140.92 (1011.14, 1270.70) | 0.0120 (0.0104, 0.0136) | 2036.96 (1769.19, 2304.73) | -0.0092 (-0.0112, -0.0072) | 69.60 (61.65, 77.55) | 0.0401 (0.0326, 0.0475) |
| Singapore | 1029.86 (910.05, 1149.67) | 0.0201 (0.0102, 0.0300) | 1826.87 (1586.53, 2067.21) | 0.0040 (-0.0120, 0.0200) | 63.57 (56.05, 71.09) | 0.0236 (0.0148, 0.0325) |
| Slovakia | 1753.22 (1490.26, 2016.18) | -0.0117 (-0.0165, -0.0069) | 3092.34 (2572.92, 3611.76) | 0.0115 (0.0049, 0.0181) | 108.23 (91.83, 124.63) | -0.0092 (-0.0159, -0.0025) |
| Slovenia | 1753.54 (1490.17, 2016.91) | -0.0082 (-0.0142, -0.0022) | 3087.95 (2569.99, 3605.91) | 0.0086 (0.0005, 0.0166) | 108.43 (92.04, 124.82) | -0.0023 (-0.0077, 0.0031) |
| Solomon Islands | 958.87 (901.83, 1015.91) | 0.0070 (0.0060, 0.0080) | 1723.87 (1596.52, 1851.22) | -0.0037 (-0.0041, -0.0034) | 58.90 (55.33, 62.47) | 0.0202 (0.0164, 0.0240) |
| Somalia | 1133.01 (1003.72, 1262.30) | 0.0220 (0.0203, 0.0237) | 2026.64 (1758.38, 2294.90) | 0.0071 (0.0052, 0.0089) | 68.96 (61.03, 76.89) | 0.0426 (0.0367, 0.0485) |
| South Africa | 1203.14 (1070.26, 1336.02) | -0.0059 (-0.0097, -0.0020) | 2138.41 (1868.08, 2408.74) | -0.0105 (-0.0120, -0.0090) | 73.68 (65.45, 81.91) | 0.0126 (0.0078, 0.0174) |
| South Sudan | 1131.34 (1002.66, 1260.02) | 0.0066 (0.0037, 0.0096) | 2029.11 (1759.62, 2298.60) | -0.0032 (-0.0069, 0.0004) | 68.78 (60.91, 76.65) | 0.0352 (0.0275, 0.0429) |
| Spain | 435.46 (392.34, 478.58) | 0.0031 (-0.0001, 0.0063) | 777.99 (693.82, 862.16) | -0.0121 (-0.0217, -0.0025) | 26.76 (24.05, 29.47) | -0.0035 (-0.0099, 0.0029) |
| Sri Lanka | 996.53 (949.05, 1044.01) | 0.0031 (0.0006, 0.0056) | 1802.21 (1741.69, 1862.73) | 0.0005 (-0.0002, 0.0012) | 61.20 (58.33, 64.07) | 0.0196 (0.0126, 0.0267) |
| Sudan | 1227.43 (1084.18, 1370.68) | 0.0094 (0.0063, 0.0125) | 2182.86 (1892.51, 2473.21) | -0.0230 (-0.0261, -0.0199) | 75.10 (66.21, 83.99) | 0.0338 (0.0270, 0.0407) |
| Suriname | 1139.34 (1007.97, 1270.71) | -0.0105 (-0.0124, -0.0086) | 2024.23 (1757.95, 2290.51) | -0.0171 (-0.0184, -0.0158) | 69.84 (61.65, 78.03) | -0.0068 (-0.0127, -0.0009) |
| Sweden | 597.42 (557.04, 637.80) | -0.0060 (-0.0095, -0.0024) | 1083.04 (998.26, 1167.82) | -0.0032 (-0.0114, 0.0050) | 36.83 (34.30, 39.36) | -0.0080 (-0.0136, -0.0023) |
| Switzerland | 467.18 (425.78, 508.58) | -0.0030 (-0.0048, -0.0011) | 841.05 (757.98, 924.12) | -0.0024 (-0.0068, 0.0019) | 28.70 (26.08, 31.32) | -0.0103 (-0.0176, -0.0030) |
| Syrian Arab Republic | 1233.84 (1090.57, 1377.11) | 0.0210 (0.0136, 0.0284) | 2190.31 (1901.38, 2479.24) | 0.0052 (-0.0028, 0.0131) | 75.66 (66.72, 84.60) | 0.0194 (0.0132, 0.0255) |
| Taiwan (Province of China) | 957.01 (905.04, 1008.98) | -0.0015 (-0.0073, 0.0043) | 1713.39 (1599.39, 1827.39) | -0.0063 (-0.0103, -0.0022) | 59.14 (55.89, 62.39) | -0.0030 (-0.0126, 0.0066) |
| Tajikistan | 1678.68 (1440.17, 1917.19) | -0.0119 (-0.0162, -0.0075) | 2974.91 (2495.84, 3453.98) | 0.0008 (-0.0039, 0.0056) | 103.17 (88.51, 117.83) | 0.0055 (-0.0002, 0.0113) |
| Thailand | 965.71 (943.09, 988.33) | 0.0014 (-0.0003, 0.0031) | 1742.06 (1692.48, 1791.64) | -0.0005 (-0.0006, -0.0003) | 59.45 (58.03, 60.87) | 0.0148 (0.0072, 0.0225) |
| Timor-Leste | 962.38 (939.52, 985.24) | 0.0112 (0.0083, 0.0141) | 1741.85 (1692.36, 1791.34) | -0.0006 (-0.0008, -0.0004) | 59.10 (57.64, 60.56) | 0.0340 (0.0277, 0.0403) |
| Togo | 1139.27 (1008.09, 1270.45) | -0.0028 (-0.0070, 0.0014) | 2027.10 (1759.88, 2294.32) | -0.0177 (-0.0194, -0.0160) | 69.32 (61.32, 77.32) | 0.0092 (-0.0018, 0.0203) |
| Tokelau | 962.29 (903.34, 1021.24) | -0.0008 (-0.0076, 0.0060) | 1718.38 (1591.98, 1844.78) | -0.0083 (-0.0133, -0.0033) | 59.20 (55.50, 62.90) | -0.0030 (-0.0124, 0.0063) |
| Tonga | 959.54 (902.37, 1016.71) | 0.0005 (-0.0007, 0.0018) | 1723.28 (1596.31, 1850.25) | -0.0016 (-0.0026, -0.0006) | 58.95 (55.41, 62.49) | -0.0009 (-0.0062, 0.0045) |
| Trinidad and Tobago | 1143.01 (1010.93, 1275.09) | -0.0087 (-0.0133, -0.0042) | 2023.49 (1758.97, 2288.01) | -0.0006 (-0.0043, 0.0032) | 70.05 (61.80, 78.30) | 0.0006 (-0.0059, 0.0071) |
| Tunisia | 1227.57 (1083.67, 1371.47) | -0.0138 (-0.0165, -0.0110) | 2174.93 (1886.98, 2462.88) | -0.0142 (-0.0189, -0.0095) | 75.71 (66.73, 84.69) | -0.0049 (-0.0112, 0.0015) |
| Turkey | 1134.64 (996.81, 1272.47) | 0.0115 (0.0089, 0.0141) | 2015.59 (1741.40, 2289.78) | -0.0081 (-0.0115, -0.0047) | 69.78 (61.16, 78.40) | 0.0233 (0.0177, 0.0290) |
| Turkmenistan | 1679.77 (1440.28, 1919.26) | -0.0066 (-0.0090, -0.0042) | 2970.70 (2492.54, 3448.86) | -0.0020 (-0.0052, 0.0011) | 103.46 (88.69, 118.23) | 0.0121 (0.0067, 0.0175) |
| Tuvalu | 957.95 (900.51, 1015.39) | 0.0061 (0.0037, 0.0085) | 1720.21 (1592.96, 1847.46) | -0.0025 (-0.0041, -0.0008) | 59.09 (55.52, 62.66) | 0.0209 (0.0135, 0.0284) |
| Uganda | 1138.47 (1008.20, 1268.74) | 0.0022 (0.0010, 0.0034) | 2029.81 (1762.42, 2297.20) | -0.0251 (-0.0270, -0.0232) | 69.67 (61.58, 77.76) | 0.0524 (0.0459, 0.0589) |
| Ukraine | 1763.52 (1520.53, 2006.51) | -0.0050 (-0.0161, 0.0060) | 3097.00 (2620.77, 3573.23) | -0.0012 (-0.0111, 0.0087) | 108.94 (93.78, 124.10) | 0.0032 (-0.0075, 0.0139) |
| United Arab Emirates | 1234.08 (1088.84, 1379.32) | 0.0086 (-0.0092, 0.0264) | 2179.10 (1891.93, 2466.27) | 0.0051 (-0.0055, 0.0156) | 75.96 (66.91, 85.01) | 0.0121 (-0.0064, 0.0307) |
| United Kingdom | 554.61 (509.13, 600.09) | 0.0173 (0.0137, 0.0210) | 996.30 (903.55, 1089.05) | 0.0134 (0.0090, 0.0177) | 34.09 (31.22, 36.96) | 0.0211 (0.0175, 0.0247) |
| United Republic of Tanzania | 1142.46 (1012.02, 1272.90) | 0.0172 (0.0158, 0.0185) | 2035.47 (1768.27, 2302.67) | -0.0084 (-0.0094, -0.0075) | 69.88 (61.82, 77.94) | 0.0784 (0.0696, 0.0872) |
| United States Virgin Islands | 1139.66 (1008.59, 1270.73) | -0.0055 (-0.0074, -0.0036) | 2018.84 (1755.49, 2282.19) | -0.0247 (-0.0273, -0.0220) | 70.12 (62.00, 78.24) | -0.0032 (-0.0096, 0.0033) |
| United States of America | 1280.95 (1108.19, 1453.71) | 0.0299 (0.0014, 0.0583) | 2273.02 (1930.88, 2615.16) | 0.0306 (0.0022, 0.0589) | 78.85 (68.01, 89.69) | 0.0269 (-0.0024, 0.0562) |
| Uruguay | 1031.81 (912.03, 1151.59) | 0.0040 (-0.0003, 0.0082) | 1824.37 (1586.58, 2062.16) | -0.0109 (-0.0160, -0.0058) | 63.65 (56.14, 71.16) | 0.0043 (-0.0022, 0.0108) |
| Uzbekistan | 1679.54 (1440.83, 1918.25) | -0.0167 (-0.0209, -0.0126) | 2974.03 (2495.61, 3452.45) | 0.0056 (0.0011, 0.0101) | 103.13 (88.42, 117.84) | -0.0044 (-0.0106, 0.0018) |
| Vanuatu | 960.80 (903.36, 1018.24) | 0.0110 (0.0095, 0.0126) | 1725.31 (1597.92, 1852.70) | -0.0005 (-0.0007, -0.0003) | 59.10 (55.52, 62.68) | 0.0172 (0.0128, 0.0217) |
| Venezuela (Bolivarian Republic of) | 1140.10 (1009.71, 1270.49) | -0.0088 (-0.0156, -0.0021) | 2029.40 (1763.16, 2295.64) | -0.0001 (-0.0036, 0.0033) | 70.27 (62.16, 78.38) | 0.0003 (-0.0081, 0.0087) |
| Viet Nam | 965.35 (942.78, 987.92) | -0.0038 (-0.0076, 0.0001) | 1741.62 (1692.20, 1791.04) | -0.0012 (-0.0013, -0.0010) | 59.38 (57.93, 60.83) | 0.0224 (0.0175, 0.0272) |
| Yemen | 1229.41 (1086.29, 1372.53) | 0.0273 (0.0258, 0.0289) | 2187.57 (1896.82, 2478.32) | 0.0021 (-0.0036, 0.0077) | 74.61 (65.96, 83.26) | 0.0366 (0.0312, 0.0421) |
| Zambia | 1143.66 (1012.76, 1274.56) | 0.0151 (0.0140, 0.0162) | 2034.60 (1767.85, 2301.35) | -0.0159 (-0.0176, -0.0141) | 69.56 (61.38, 77.74) | 0.0513 (0.0428, 0.0598) |
| Zimbabwe | 1142.66 (1011.99, 1273.33) | 0.0021 (0.0002, 0.0040) | 2034.75 (1767.62, 2301.88) | -0.0049 (-0.0072, -0.0026) | 70.06 (61.99, 78.13) | 0.0070 (0.0009, 0.0130) |
| DALYs, disability-adjusted life-years; ASR, age-standardized rate; EAPC, estimated annual percentage change; UI, uncertainty interval. | | | | | | |

**Supplementary Figure 1. The global incidence of urticaria in children and adolescents aged <20 years in 204 countries and territories. (A) Age-standardised incidence rate in 2021. (B) Estimated annual percentage change (EAPC) in incidence from 1990 to 2021.**

**
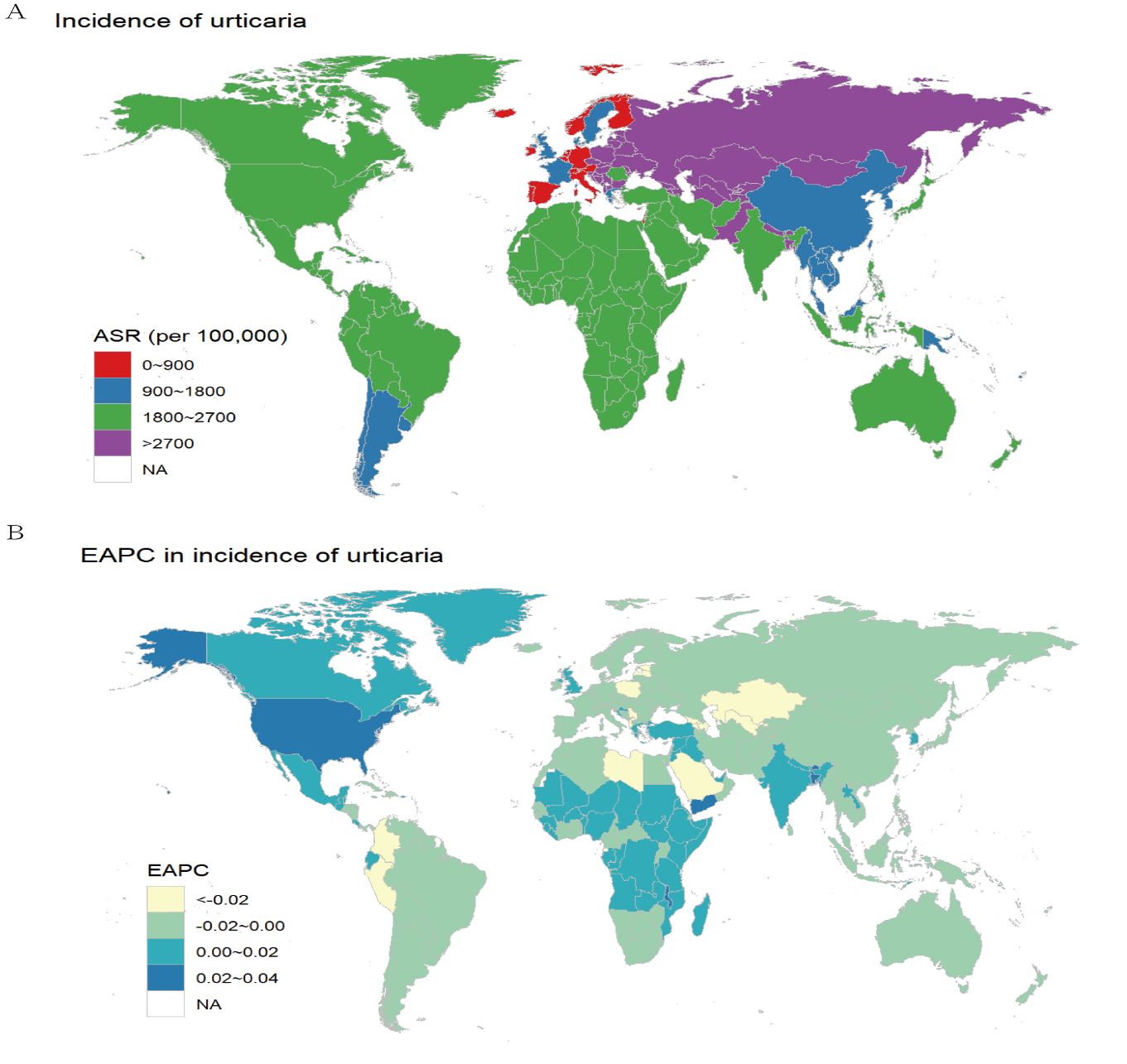
**

**Supplementary Figure 2. The global DALYs of urticaria in children and adolescents aged <20 years in 204 countries and territories. (A) Age-standardised DALYs rate in 2021. (B) Estimated annual percentage change (EAPC) in DALYs from 1990 to 2021.**

**
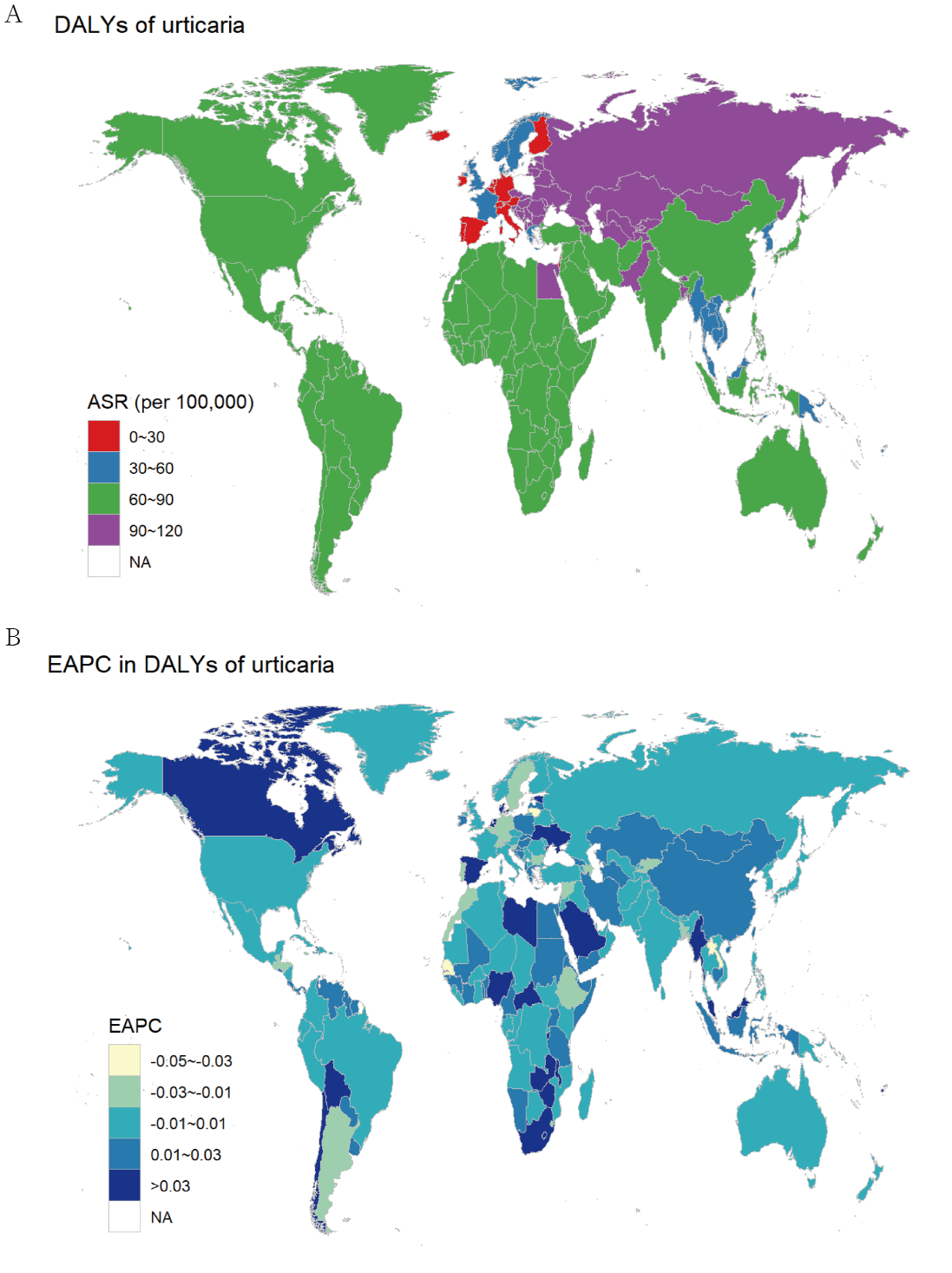
**
